# CircPPFIA2 drives prostate cancer progression and enzalutamide resistance by sponging miR-646 and miR-1200 to upregulate ETS1

**DOI:** 10.1038/s41420-025-02904-z

**Published:** 2025-12-08

**Authors:** Yiyou Mao, Qu Leng, Jun Wu, Wenbin Chen, Chunxi Lin, Zhihai Deng, Qiang Shen, Jun Zou, Zining Long, Yiyuan Zhan, Shilong Cheng, Zhongjie Chen, Rui Zhou, Jiaxing Wang, Hangyang Peng, Yangbai Lu, Yilan Huang, Chenglu Li, Aihua Cai, Jingyan Xu, Hongxing Huang, Dongmei Jiang, Xiangming Mao, Daojun Lv

**Affiliations:** 1 Department of Urology, Guangdong Provincial Key Laboratory of Major Obstetric Diseases, Guangdong Provincial Clinical Research Center for Obstetrics and Gynecology, The Third Affiliated Hospital, Guangzhou Medical University, Guangzhou, 510150 China; 2 Department of Urology, Zhujiang Hospital, Southern Medical University, Guangzhou, 510280 China; 3 Department of Urology, Zhongshan City People’s Hospital, Zhongshan, Guangdong 528403 China; 4 Department of Urology, Shanghai Ninth People’s Hospital, Shanghai Jiao Tong University School of Medicine, Shanghai, 200011 China; 5 The Third Clinical College, Guangzhou Medical University, Guangzhou, 511495 China; 6 Department of Emergency, Guangdong Provincial Key Laboratory of Major Obstetric Diseases, Guangdong Provincial Clinical Research Center for Obstetrics and Gynecology, The Third Affiliated Hospital, Guangzhou Medical University, Guangzhou, 510150 China; 7 The Second Clinical College, Guangzhou Medical University, Guangzhou, 511495 China; 8 Department of Pathology, The First Affiliated Hospital, Guangzhou Medical University, Guangzhou, 510120 China; 9 Guangdong Provincial Key Laboratory of Urological Diseases, Guangzhou Medical University, Guangzhou, 510275 China

**Keywords:** Tumour biomarkers, Prostate cancer

## Abstract

Prostate cancer (PCa) represents a leading cause of cancer-related morbidity in men worldwide, necessitating deeper insights into its molecular drivers. Circular RNAs (circRNAs) are increasingly recognized as key regulatory molecules in carcinogenesis; however, their functional significance in PCa pathogenesis and treatment resistance remains incompletely defined. Here, we identify circPPFIA2 as a novel clinically relevant oncogenic circRNA with dual roles in PCa progression and therapeutic resistance. CircPPFIA2 is markedly upregulated in PCa clinical specimens and cell lines. Through gain- and loss-of-function experiments in both cell-based and animal models, we established that circPPFIA2 drives oncogenic phenotypes by enhancing tumor cell proliferation, migratory capacity, and resistance to enzalutamide therapy. Mechanistic investigations revealed that circPPFIA2 functions as a competitive endogenous RNA (ceRNA), simultaneously sequestering tumor-suppressive miR-646 and miR-1200. This miRNA sponge activity facilitates post-transcriptional upregulation of ETS1, a critical effector of androgen receptor signaling and treatment resistance. This molecular interplay establishes the circPPFIA2/miR-646/miR-1200/ETS1 axis as a central driver of PCa progression and therapy resistance. To functionally validate this finding, we employed lipid nanoparticle (LNP)-mediated co-delivery of si-circPPFIA2 and enzalutamide, which effectively restored drug sensitivity and inhibited tumor growth in resistant PCa models. Our findings highlight circPPFIA2 as both a prognostic biomarker and a promising therapeutic target for advanced PCa, providing a rationale for developing circRNA-directed therapies to overcome treatment resistance.

## Introduction

Prostate cancer (PCa) ranks as the second most prevalent malignancy and a leading cause of cancer-associated death among men globally, with an estimated annual incidence exceeding 1.5 million cases and approximately 397,000 fatalities [1–3]. The therapeutic landscape for advanced PCa continues to be dominated by androgen deprivation therapy (ADT), which mechanistically targets androgen receptor (AR) signaling cascades [4, 5]. However, nearly all patients eventually develop castration-resistant prostate cancer (CRPC) through adaptive cellular mechanisms that bypass AR dependency. The historical research paradigm in CRPC pathogenesis has predominantly centered on androgen signaling axis alterations, leading to the development of second-generation AR pathway inhibitors, including abiraterone and enzalutamide (Enza) [6, 7]. These targeted agents initially demonstrated marked clinical benefits, significantly improving progression-free survival and overall survival outcomes [8, 9]. Unfortunately, the therapeutic durability of these interventions remains constrained by the inevitable emergence of acquired resistance mechanisms driven by AR splice variants (e.g., AR-V7), compensatory signaling pathways, or transcriptional bypass severely compromises long-term therapeutic efficacy, which means that no effective treatment is available for CRPC patients [10]. This persistent clinical challenge underscores the urgent need to delineate new strategies for reducing drug resistance to next-generation AR-targeted therapy, which may improve the prognosis of CRPC patients.

Circular RNAs (circRNAs), a novel class of endogenous single-stranded non-coding RNAs generated through back-splicing of precursor messenger RNA (pre-mRNA) transcripts [11–13], have emerged as pivotal regulators in cancer pathophysiology. Distinguished by their circular architecture and evolutionary conservation, these single-stranded RNA molecules exhibit unique biostability through resistance to exonucleolytic degradation, establishing them as promising candidates for therapeutic intervention [14, 15]. Their functional repertoire spans critical cancer hallmarks, including tumorigenesis initiation, metastatic progression, and therapeutic resistance, with accumulating evidence revealing widespread dysregulation of circRNA networks across multiple malignancies [16]. In PCa, mechanistic studies have delineated specific circRNA-mediated regulatory axes governing tumor progression. For instance, the oncogenic hsa_circ_0003258 orchestrates metastatic dissemination through forming ribonucleoprotein complexes with IGF2BP3 while simultaneously sequestering tumor-suppressive miR-653-5p [17]. In contrast, hsa_circ_0063329 functions as a tumor-suppressive decoy RNA by competitively binding miR-605-5p, thereby de-repressing TGIF2 expression to inhibit malignant proliferation and metastatic spread [18]. These paradigm-shifting findings underscore the emerging recognition of circRNAs as master regulators of PCa progression. Notwithstanding these advances, the comprehensive circRNA interactome in PCa remains poorly mapped, particularly regarding its contribution to enzalutamide resistance.

In this study, we characterized circPPFIA2 (circBase ID: hsa_circ_00099343), a previously unstudied circRNA derived from exon circularization of the PPFIA2 locus. The profile and closed-loop conformation of circPPFIA2 were rigorously validated in PCa cell lines and clinical specimens. Through comprehensive functional genomics approaches, we systematically interrogated its oncogenic role in PCa progression and dissected its molecular mechanism of action. Our work positions circPPFIA2 as both a prognostic biomarker and a therapeutically actionable target in advanced PCa, providing novel therapeutic strategies for circRNA-directed interventions to circumvent treatment resistance.

## Results

### The screening and characteristics of circPPFIA2 in PCa

A genome-wide CRISPR/Cas9 loss-of-function screen previously implicated heterogeneous nuclear ribonucleoprotein L (HNRNPL) as a regulator of circRNA biogenesis in PCa through modulation of RNA splicing dynamics [19]. To delineate its regulatory landscape, we reanalyzed circRNA expression profiles from HNRNPL-knockdown versus control LNCaP cells (GSE72840 dataset). Among 20 significantly upregulated circRNAs (Supplementary Fig. S1A), hsa_circ_0099343 (annotated as circPPFIA2) emerged as the lead candidate based on two criteria: (1) its host gene PPFIA2 exhibited the highest baseline expression in PCa cells, suggesting robust transcriptional activity conducive to circRNA biogenesis (Supplementary Fig. S1B); (2) conserved HNRNPL binding motifs were identified within flanking introns of PPFIA2, suggesting direct splicing regulation. To interrogate the molecular drivers of circPPFIA2 formation, RNA pull-down assays coupled with silver staining and western blot identified HNRNPL as a direct interactor of circPPFIA2 (Supplementary Fig. S1C, D). RIP assays further confirmed significant enrichment of circPPFIA2 in HNRNPL-bound ribonucleoprotein complexes (Supplementary Fig. S1E, F). Intriguingly, siRNA-mediated HNRNPL knockdown paradoxically increased circPPFIA2 levels while leaving linear PPFIA2 mRNA expression unaltered (Supplementary Fig. S1G, H), suggesting a suppressive role for HNRNPL in circPPFIA2 biogenesis. These findings position circPPFIA2 as a key HNRNPL-repressed circRNA with potential oncogenic relevance in PCa.

**Fig. 1:**
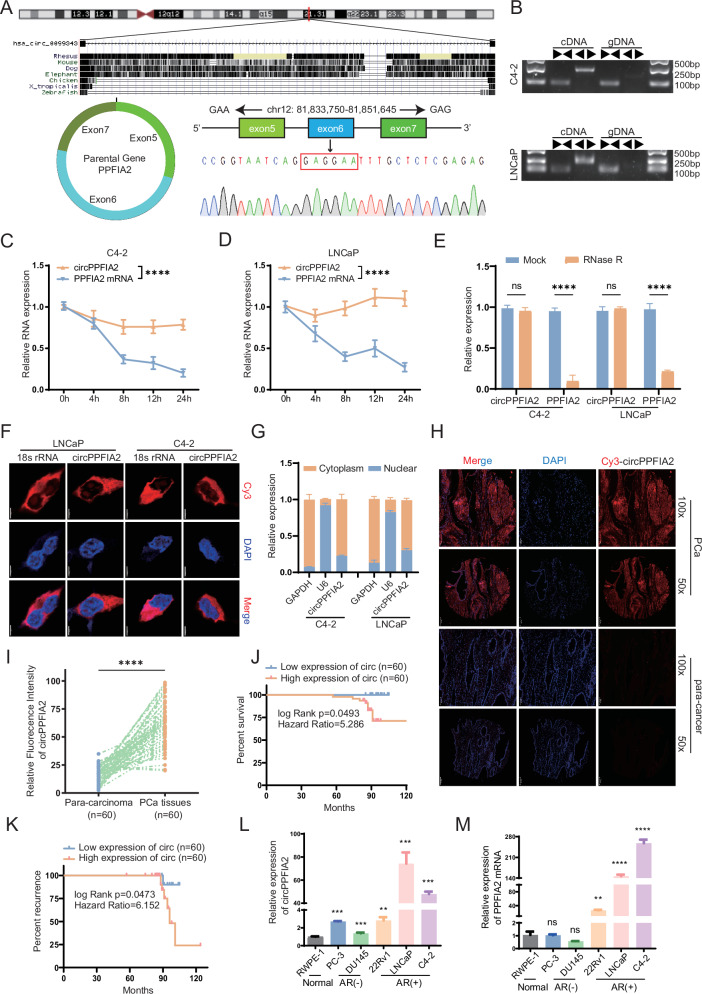
CircPPFIA2 is upregulated and predicts adverse clinical Outcomes of PCa. **A** Schematic of circPPFIA2 biogenesis from the PPFIA2 locus, validated by Sanger sequencing. **B** Divergent/convergent primer amplification confirms circularity in cDNA (vs. gDNA) via qRT-PCR and agarose gel electrophoresis. **C**–**E** circPPFIA2 resistance to RNase R and Actinomycin D validates its closed-loop structure. **F** RNA-FISH reveals cytoplasmic circPPFIA2 localization (DAPI: nuclei; 18S rRNA: cytoplasmic control). Scale bar: 5 μm. **G** Subcellular fractionation with qRT-PCR confirms circPPFIA2 cytoplasmic enrichment (GAPDH: cytoplasmic control; U6: nuclear control). RNA-FISH demonstrates circPPFIA2 overexpression in PCa tissues. Scale bars: 100 μm (**H**), 200 μm (**I**). TMA cohort: *n* = 60 cases; statistics as indicated. Kaplan-Meier analysis correlates high circPPFIA2 with reduced overall survival (**J**) and accelerated biochemical recurrence (**K**). **L**, **M** The expression levels of circPPFIA2 in 5 PCa cell lines compared with RWPE-1 cell line. Data are presented as mean ± SD (*n* = 3 biological replicates); *p < 0.05, **p < 0.01, ***p < 0.001, ****p < 0.0001.

CircPPFIA2 (genomic location: chr12: 81,833,750-81,851,645; circBase ID: has_circ_00099343) was generated from the exon5-7 of PPFIA2 gene, as confirmed by Sanger sequencing of the head-to-tail junction (Fig. 1A). To further confirm the circular structure, we designed divergent and convergent PCR primers, and found that circPPFIA2 was only amplified by divergent primers with cDNA (Fig. 1B). Then, biochemical characterization demonstrated marked stability of circPPFIA2 compared to its linear counterpart, evidenced by prolonged half-life (>24 h for circRNA vs. <8 h for linear RNA) under Actinomycin D transcriptional blockade (Fig. 1C, D) and resistance to RNase R exonuclease digestion (Fig. 1E). Subcellular fractionation coupled with RNA-FISH revealed predominant cytoplasmic localization of circPPFIA2 in PCa cells (Fig. 1F, G), suggesting potential roles in post-transcriptional regulation or miRNA sponging a hallmark of cytoplasmic circRNA functionality. Collectively, these data establish the circular nature of circPPFIA2 and confirm its presence in PCa cells.

### Elevated circPPFIA2 correlated with aggressive clinical features and poor clinical outcomes in PCa patients

To delineate the expression landscape and clinical significance of circPPFIA2 in PCa, we performed RNA fluorescence in situ hybridization (FISH) on a tissue microarray (TMA) comprising 60 clinically annotated PCa specimens. Comparative analysis revealed robust circPPFIA2 overexpression in malignant lesions compared to matched adjacent non-neoplastic tissues (*p* < 0.0001; Fig. 1H, I, and Supplementary Fig. S1I). Clinicopathological stratification demonstrated a statistically significant association between elevated circPPFIA2 levels and advanced Gleason scores (p = 0.012), while no correlation was observed with patient age or tumor stage (Table 1). Survival analysis further established the prognostic relevance of circPPFIA2, with Kaplan-Meier curves showing markedly accelerated biochemical recurrence (HR = 6.152, *p* = 0.0473) and reduced overall survival (HR = 5.286, *p* < 0.0493) in high-expression cohorts (Fig. 1J, K). This tumor-specific expression pattern was recapitulated in PCa cellular models, where circPPFIA2 exhibited robust upregulation across multiple cell lines compared to normal prostate epithelial cells (RWPE-1). Notably, AR-positive cell lines (LNCaP, C4-2) showed the most dramatic elevation ( > 40-fold increase vs. controls), while AR-negative models (DU145, PC3) maintained moderate overexpression (Fig. 1L). Strikingly, linear PPFIA2 mRNA displayed a parallel but less pronounced trend (Fig. 1M), suggesting circPPFIA2 accumulation occurs through post-transcriptional regulatory mechanisms independent of its parental gene. In summary, these findings position circPPFIA2 as a biomarker independently predictive of aggressive disease progression and therapeutic resistance in PCa.

**Table 1:** The correlation between circPPFIA2 expression and clinicopathological characteristics was analyzed in PCa by FISH (60 PCa cases with matched para-carcinoma tissues).

	Group	*N*	circPPFIA2		χ^2^	*P* value^a^
			Low (n, %)	High (n, %)		
Type	Para-carcinoma	60	60 (100.00)	0 (0.00)	77.260	<0.01
	Adenocarcinoma	60	13 (21.67)	47 (78.33)		
Age	≤65	28	4 (14.29)	24 (85.71)	1.685	0.194
	> 65	32	9 (28.13)	23 (71.88)		
Gleason score	≤6	11	6 (54.55)	5 (45.45)	6.371	0.012
	≥7	49	7 (14.29)	42 (85.71)		

TMA sections were evaluated by RNA FISH and cases were dichotomized into high and low circPPFIA2 expression according to the H-score threshold. Associations with clinicopathological variables were analyzed using Pearson’s χ² test (two-sided), with p < 0.05 considered significant.

*PCa* prostate cancer, *TMA* tissue microarray, *FISH* fluorescence in situ hybridization.

^a^*p* value from χ² test.

### CircPPFIA2 facilitates PCa progression via cell cycle dysregulation, apoptotic evasion and EMT activation in vitro

**Fig. 2:**
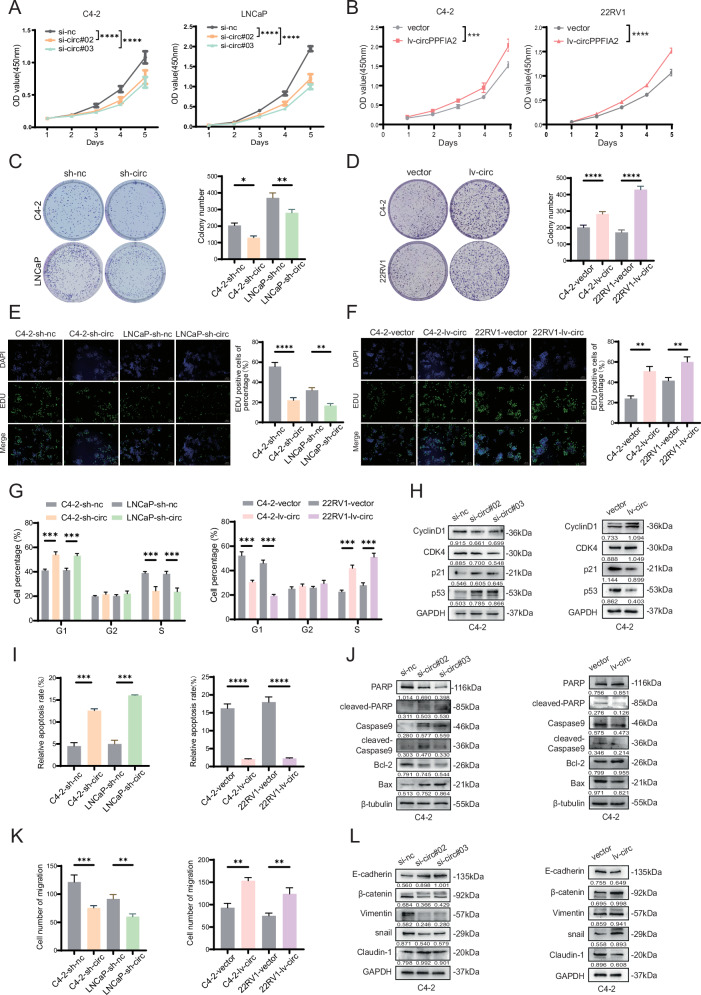
CircPPFIA2 promotes proliferation and migration of PCa cells in vitro. The proliferative capacity of PCa cells was assessed using CCK-8 (**A**, **B**), colony formation (**C**, **D**), and EdU incorporation assays (**E**, **F**). These experiments were performed on C4-2, LNCaP and 22Rv1 cells with either circPPFIA2 knockdown or overexpression. The scale bar in relevant images is 50 μm. **G** Flow cytometry was employed to analyze the cell cycle distribution in PCa cells with circPPFIA2 knockdown and overexpression. **H** WB analysis was used to evaluate the expression levels of cell cycle associated proteins in these cells. **I** Flow cytometry was utilized to detect the apoptosis rate of PCa cells after circPPFIA2 knockdown and overexpression. **J** WB analysis was also carried out to assess the expression of apoptosis - associated proteins. **K** Transwell migration assay was conducted to evaluate the migration ability of PCa cells with circPPFIA2 knockdown and overexpression. **L** WB analysis was performed to examine the expression levels of EMT biomarkers in these cells. Data are presented as mean ± SD (*n* = 3 biological replicates). Two-tailed tests with Bonferroni correction were used. *p < 0.05, **p < 0.01, ***p < 0.001, ****p < 0.0001.

To investigate the oncogenic role of circPPFIA2 in PCa, we performed bidirectional functional modulation through siRNA-mediated knockdown in AR-positive C4-2 and LNCaP cells and lentiviral overexpression in C4-2 and 22Rv1 models. Subsequently, we used qPCR confirmed efficient circPPFIA2 suppression or induction without altering linear PPFIA2 mRNA levels, indicating manipulation specificity (Supplementary Fig. S2A–C). Then, functional assays revealed that circPPFIA2 knockdown significantly inhibited proliferation in C4-2 and LNCaP cells, as evidenced by reduced viability (CCK-8 tests), impaired clonogenicity and suppressed DNA synthesis (EdU assays), whereas overexpression accelerated proliferation in C4-2 and 22Rv1 (Fig. 2A–F). In addition, flow cytometry analysis demonstrated that circPPFIA2 silencing induced G1/S-phase arrest, and Western blot revealed that circPPFIA2 depletion upregulated p53/p21 and downregulated Cyclin D1/CDK4, whereas overexpression exerted reciprocal effects (Fig. 2G, H, and Supplementary Fig. S2D, E). Mechanistically, flow cytometry showed that circPPFIA2 silencing stimulated apoptosis, and this apoptotic regulation was associated with increased Bax and Cleaved PARP/Caspase9 levels alongside decreased Bcl-2 upon circPPFIA2 depletion, with overexpression conversely modulating these markers (Fig. 2I, J, and Supplementary Fig. 2F, G). Following this, Transwell assays and EMT marker analysis demonstrated that circPPFIA2 knockdown suppressed migration and promoted an epithelial phenotype in PCa cells, while its overexpression enhanced migratory capacity and induced mesenchymal marker expression (Fig. 2K, L, and Supplementary Fig. S2H, I). However, under Matrigel-coated conditions, circPPFIA2 perturbation produced modest, model-dependent differences in invasion that did not reach uniform statistical significance across C4-2, LNCaP, and 22Rv1 (Supplementary Fig. S2J, K); we therefore present these data as exploratory. Collectively, these findings establish circPPFIA2 as a multimodal oncogenic driver that coordinates proliferative signaling, apoptotic resistance, and metastatic competence in PCa.

### CircPPFIA2 promotes the growth and metastasis of PCa cells in vivo

**Fig. 3:**
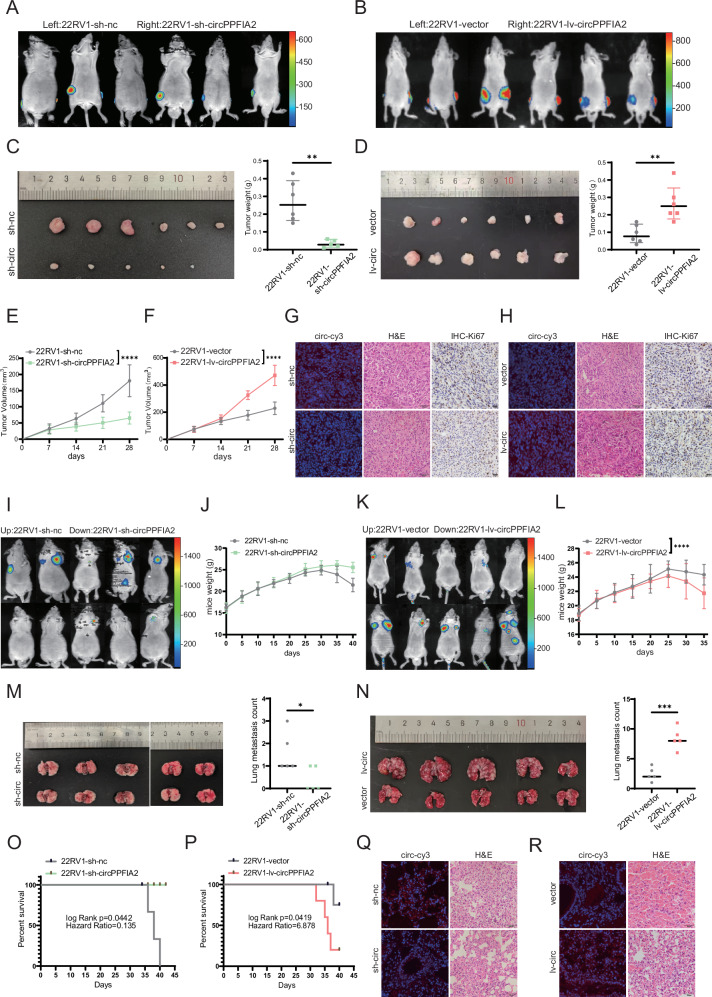
CircPPFIA2 Drives Proliferation and Metastasis of PCa Cells in Vivo. **A**, **B** Fluorescence imaging of subcutaneous xenograft tumors derived from 22Rv1 cells transfected with negative control (NC) or circPPFIA2-modulating constructs (sh-circPPFIA2 knockdown or lv-circPPFIA2 overexpression). *n* = 6 mice per group. **C**, **D** Macroscopic analysis and tumor weights demonstrate circPPFIA2-dependent growth modulation. **E**–**H** Tumor volume kinetics and histopathological characterization (RNA-FISH, H&E, Ki-67) of subcutaneous tumors. Scale bar: 50 μm. **I**–**N** Bioluminescence imaging and gross observation of lung metastases, with circPPFIA2 knockdown reducing metastatic burden and overexpression enhancing nodule formation. *n* = 5 mice per group. **O**–**R** Kaplan-Meier survival curves confirm circPPFIA2 knockdown extends median survival, while overexpression correlates with mortality acceleration. Metastatic lesions exhibit circPPFIA2-dependent molecular profiles (RNA-FISH, H&E, Ki-67). Data are presented as mean ± SD (*n* = 3 biological replicates). Two-tailed tests with Bonferroni correction were used; *p < 0.05, **p < 0.01, ***p < 0.001, ****p < 0.0001.

To investigate the oncogenic role of circPPFIA2 in PCa progression in vivo, we established subcutaneous xenograft models using 22Rv1 cells stably expressing sh-circPPFIA2 or control shRNA, alongside circPPFIA2-overexpressing or vector-control cells. Within 30 days post-inoculation, circPPFIA2 knockdown significantly attenuated tumor growth, with sh-circPPFIA2 xenografts exhibiting smaller tumor volumes and reduced weights compared to controls, while circPPFIA2-overexpressing 22Rv1 cells demonstrated accelerated tumor growth (Fig. 3A–F). Histopathological analysis (H&E staining) confirmed malignant architecture in all cohorts, while IHC revealed markedly reduced Ki67 proliferation indices in sh-circPPFIA2 tumors and elevated Ki67 in circPPFIA2-overexpressing xenografts (Fig. 3G, H). To assess metastatic potential, we employed a tail vein metastasis model. Mice injected with sh-circPPFIA2 cells exhibited fewer lung metastatic nodules and minimal peritoneal dissemination compared to controls (Fig. 3I–N). Survival analysis further demonstrated that circPPFIA2 knockdown extended median survival of mice (log-rank *p* = 0.0442; Fig. 3O). In contrast, circPPFIA2 overexpression exacerbated metastatic burden and reduced median survival (log-rank *p* = 0.0419; Fig. 3P). Histopathological characterization of lung metastases via H&E staining confirmed invasive tumor foci, and RNA FISH further validated circPPFIA2 downregulation in sh-circPPFIA2 lung lesions and overexpression in circPPFIA2-enhanced metastases (Fig. 3Q, R). These findings conclusively establish circPPFIA2 as a critical regulator of PCa growth and metastasis in vivo, with bidirectional modulation of its expression directly correlating with tumor aggressiveness, metastatic potential, and survival outcomes, underscoring its therapeutic relevance in advanced PCa.

### CircPPFIA2 serves as a functional miR-646/miR-1200 sponge to drive oncogenic signaling

**Fig. 4:**
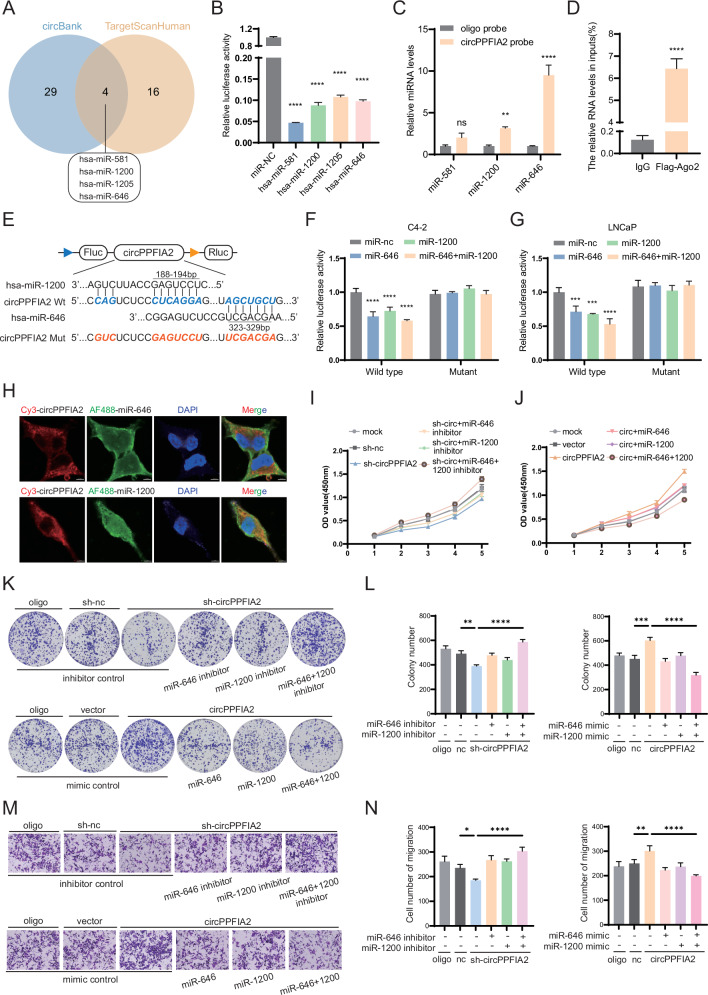
CircPPFIA2 Facilitates PCa Progression via miR-646/miR-1200 Sponging. **A** Venn diagram identifies miR-646, miR-1200, and miR-581 as top candidate miRNAs targeting circPPFIA2. **B** Luciferase reporter assays validate miR-646/miR-1200-mediated suppression of circPPFIA2 activity in HEK-293T cells. **C** Biotin-tagged circPPFIA2 or control probes were used in pull-down assay of miRNAs in C4-2 cell. **D** qRT-PCR analysis of circPPFIA2 enrichment in Flag-Ago2 immunoprecipitates, with IgG as the negative control. *n* = 3 biological replicates; *n* = 3 biological replicates; values are shown as % input normalized to IgG. **E** Schematic of circPPFIA2 reporters showing conserved miR-1200 (bp 188–194) and miR-646 (bp 323–329) binding sites; the mutant contains point mutations at these miRNA-binding sites. **F**, **G** Dual-luciferase assays showing that co-transfection of miR-1200/miR-646 mimics reduced WT reporter activity; this effect was abolished in the mutant reporter with miRNA-binding-site mutations. *n* = 3 biological replicates; values are firefly normalized to Renilla and to miR-NC. **H** RNA-FISH confirms cytoplasmic co-localization of circPPFIA2 with miR-646/miR-1200. Scale bar: 5 μm. **I**–**N** Rescue experiments demonstrate miR-646/miR-1200 inhibitors reverse circPPFIA2 silencing effects, whereas mimics counteract circPPFIA2-driven oncogenicity. Scale bar: 50 μm. Data are presented as mean ± SD (*n* = 3 biological replicates). Two-tailed tests with Bonferroni correction were used; *p < 0.05, **p < 0.01, ***p < 0.001, ****p < 0.0001.

Considering the predominant cytoplasmic enrichment of circPPFIA2 in PCa cells (Fig. 1M), we hypothesized a miRNA-sponge mechanism [20, 21]. Using circBank and TargetScan, we predicted four candidates—miR-581, miR-1200, miR-646, and miR-1205 (Fig. 4A). A HEK-293T dual-luciferase screen showed that miR-581, miR-1200, and miR-646 directly repressed circPPFIA2 reporter activity upon co-transfection (Fig. 4B). In C4-2 cells, biotinylated circPPFIA2 pull-down selectively enriched miR-646 and miR-1200 compared to a scrambled control (Fig. 4C). RIP with FLAG-Ago2 further demonstrated circPPFIA2 association with the RNA-induced silencing complex (Fig. 4D, and Supplementary Fig. S3A). Reporter assays with wild-type and binding-site-mutant circPPFIA2 confirmed sequence-specific suppression by miR-1200/miR-646 in C4-2 cells, which was abolished by site mutation (Fig. 4E–G). Dual-probe RNA-FISH showed cytoplasmic co-localization of circPPFIA2 with miR-646/miR-1200 (Fig. 4H). Collectively, these data demonstrate circPPFIA2 acting as a sponge for miR-646 and miR-1200 in PCa.

### MiR-646/miR-1200 suppress PCa progression and reverses the tumor oncogenic role of circPPFIA2

To elucidate the functional role of miR-646/miR-1200 in PCa, we performed gain- and loss-of-function experiments. CCK-8, colony formation and Transwell assays demonstrated that miR-646/miR-1200 mimics significantly suppressed prostate cancer cell proliferation and migration, whereas their inhibitors enhanced both processes, highlighting the bidirectional regulatory roles of these miRNAs (Supplementary Fig. S3B–F). Rescue experiments further validated the antagonistic relationship between circPPFIA2 and these miRNAs, overexpression of miR-646/miR-1200 completely abrogated circPPFIA2-induced proliferation and migration in C4-2 cells, whereas miRNA inhibition reversed the tumor-suppressive effects of circPPFIA2 knockdown (Fig. 4I−N). Together, these results define miR-646/miR-1200 as tumor-suppressive regulators that directly antagonize the oncogenic functions of circPPFIA2, positioning them as promising therapeutic targets for attenuating PCa progression through disruption of the circRNA-miRNA regulatory axis.

### CircPPFIA2 activates ETS1-driven oncogenic signaling via sponging miR-646/miR-1200 in PCa

**Fig. 5:**
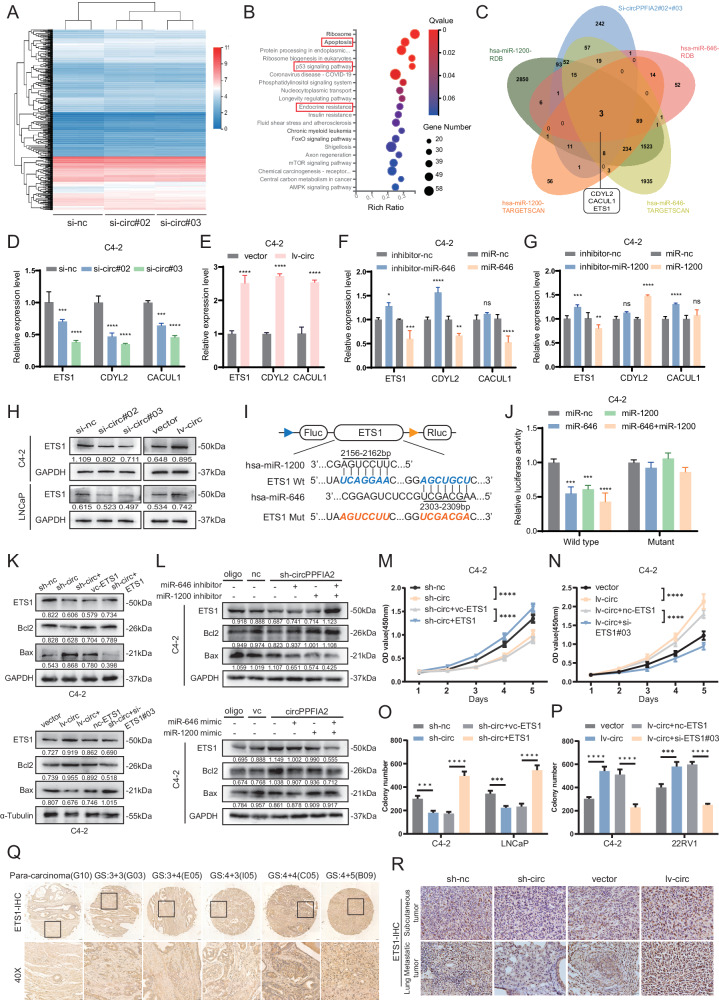
CircPPFIA2 Promotes Oncogenic Signaling via miR-646/miR-1200 Mediated ETS1 Regulation. **A**, **B** RNA-seq of circPPFIA2-silenced C4-2 cells identified 985 DEGs; KEGG enrichment highlighted apoptosis and p53 pathways. **C** ETS1 emerged as the shared target of miR-646/miR-1200. **D**–**G** qRT-PCR: circPPFIA2 knockdown decreased ETS1 and was rescued by miR-646/miR-1200 inhibitors; circPPFIA2 overexpression increased ETS1 and was blunted by the corresponding mimics. **H** WB confirmed circPPFIA2-dependent modulation of ETS1 protein. **I** Schematic of ETS1 3’UTR reporters with miR-1200 (bp 2156-2162) and miR-646 (bp 2303-2309) binding sites; the mutant contains point mutations at these miRNA-binding sites. **J** Dual-luciferase assays demonstrating direct targeting of the ETS1 3’UTR: co-transfection reduced WT reporter activity, which was abolished in the mutant reporter with miRNA-binding-site mutations. **K**, **L** Rescue assays: ETS1 reconstitution in sh-circ cells restored Bcl2 and reduced Bax, whereas ETS1 knockdown reversed circPPFIA2-driven anti-apoptotic changes. Functional rescue: ETS1 overexpression restored proliferation suppressed by circPPFIA2 knockdown (CCK-8, **M**; colony formation, **O**); conversely, ETS1 silencing abrogated lv-circ-induced proliferation (**N**, **P**). **Q** IHC of clinical specimens shows ETS1 upregulation with higher Gleason scores. **R** Subcutaneous and metastatic xenografts recapitulated ETS1/Bcl2/Bax dysregulation. Scale bars: 100 μm (**Q**), 20 μm (**R**). Data are presented as mean ± SD (n = 3 biological replicates). Two-tailed tests with Bonferroni correction were used; *p < 0.05, **p < 0.01, ***p < 0.001.

MicroRNAs (miRNAs) are critical post-transcriptional regulators of oncogenic signaling networks. Building on our discovery that circPPFIA2 functions as a molecular sponge for miR-646 and miR-1200, we sought to delineate their downstream targets mediating PCa progression. RNA-seq analysis of C4-2 cells transfected with si-circPPFIA2 identified 482 upregulated and 503 downregulated genes (Fig. 5A). KEGG analysis revealed enrichment in apoptosis, p53 signaling, and endocrine resistance pathways (Fig. 5B, and Supplementary Fig. S4A), while Gene ontology (GO) analysis showed predominant cytoplasmic localization of differentially expressed genes (Supplementary Fig. S4B, C). Integrative bioinformatics analysis (TargetScan, miRDB) identified three candidate mRNAs (ETS1, CDYL2, CACUL1) whose predicted binding sites overlapped with transcriptomic downregulation in circPPFIA2-silenced C4-2 cells (Fig. 5C). Functional validation revealed that ETS1, CDYL2 and CACUL1 expression was positively regulated by circPPFIA2 levels, as evidence by knockdown of circPPFIA2 suppressed their expression, while overexpression enhanced mRNA levels (Fig. 5D, E, and Supplementary Fig. S4D, E). Strikingly, we further modulated miR-646/miR-1200 levels via mimics or inhibitors and found that ETS1 exhibiting the most pronounced changes among these three mRNAs (Fig. 5F, G, and Supplementary Fig. S4F, G). Subsequently, Western blot analysis confirmed that ETS1 protein levels are regulated by circPPFIA2(Fig. 5H). To validate direct targeting, luciferase reporter assays demonstrated that miR-646/miR-1200 mimics reduced wild-type ETS1 3’-UTR luciferase activity, while mutant ETS1 3’-UTR (binding site deletions) remained unaffected (Fig. 5I, J, and Supplementary Fig. S4H). These results confirm that miR-646/miR-1200 directly repress ETS1 via conserved 3’-UTR interactions.

Next, we hypothesized that circPPFIA2 drives PCa progression by sponging miR-646 and miR-1200 to derepress ETS1, a master transcriptional effector of oncogenic signaling. To delineate the functional contribution of ETS1 to this pathway, we validated ETS1-targeting siRNA and overexpression plasmids through qRT-PCR and Western blot analyses (Supplementary Fig. S5A–D). Western blot profiling demonstrated that circPPFIA2-driven upregulation of ETS1 and Bcl2 was effectively reversed by miR-646/miR-1200 mimic co-transfection (Fig. 5K, L, and Supplementary Fig. S5E, F). Rescue experiments conclusively established the axis dependency, circPPFIA2 overexpression robustly enhanced PCa cell proliferation and migration, whereas ETS1 knockdown fully abrogated these effects (Fig. 5M−P, and Supplementary Fig. S5G–M), confirming ETS1 as the pivotal downstream effector of circPPFIA2-mediated oncogenicity. Clinical immunohistochemical analyses demonstrated marked upregulation of ETS1 protein in PCa tissues compared to benign controls (Fig. 5Q), aligning with its proposed oncogenic role. Consistent with these findings, immunohistochemistry of tumor tissues revealed that circPPFIA2 silencing significantly reduced ETS1 and Bcl2 expression (Fig. 5R and Supplementary Fig. S5N), whereas circPPFIA2 overexpression elevated ETS1 levels, reinforcing their causal relationship. These integrated results establish the circPPFIA2/miR-646/miR-1200/ETS1 axis as a central oncogenic driver in PCa, wherein circPPFIA2 acts as a molecular sponge to liberate ETS1 from miRNA-mediated repression, unleashing its tumor-promoting transcriptional programs. This mechanistic paradigm not only deepens our understanding of circRNA-driven carcinogenesis but also positions the circPPFIA2-ETS1 axis as a promising therapeutic target for disrupting oncogenic signaling networks in advanced PCa.

### CircPPFIA2 drives enzalutamide resistance in PCa through ETS1

**Fig. 6:**
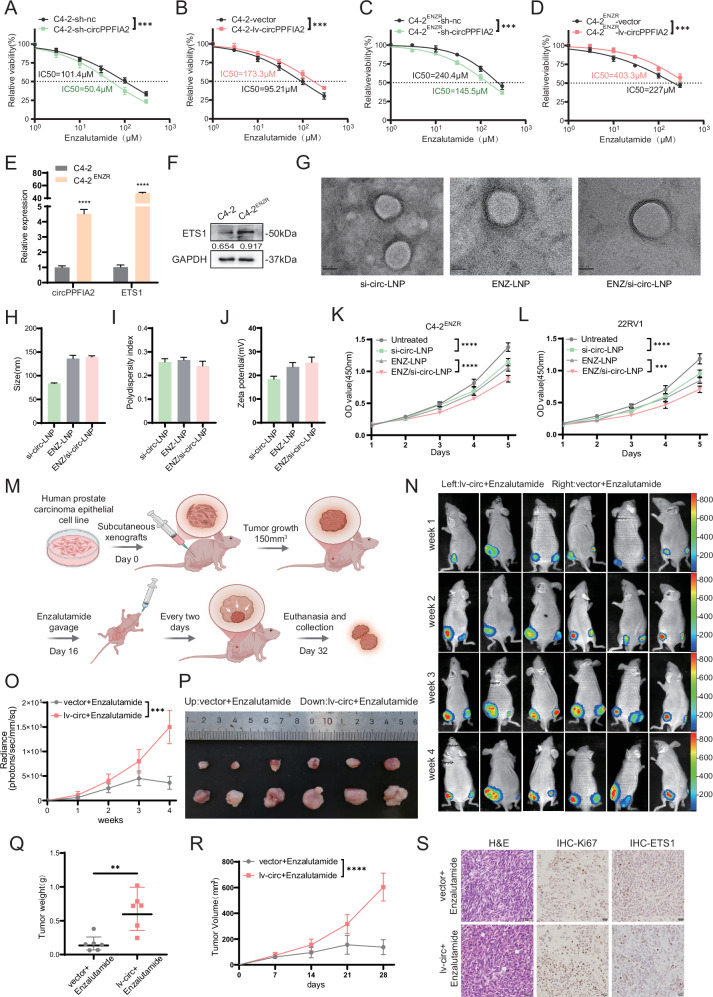
circPPFIA2 modulates enzalutamide sensitivity in PCa. **A**–**D** Dose-response curves and IC50 quantification demonstrating that circPPFIA2 knockdown sensitized parental C4-2 cells to enzalutamide, while circPPFIA2 overexpression conferred resistance in enzalutamide-refractory C4-2 cells. **E**, **F** Enzalutamide-resistant C4-2 cells exhibit upregulated circPPFIA2 and ETS1 compared to parental cells, validated by qRT-PCR and Western blot. **G** Representative TEM images of si-circ-LNP, ENZ-LNP, and ENZ/si-circ-LNP. The scale bar is 50 nm. Particle size (**H**), PDI (**I**) and zeta potential (**J**) of LNPs. **K**, **L** CCK-8 assays in C4-2ENZR and 22Rv1 cells after treatment with the indicated LNPs. **M** Schematic of the subcutaneous xenograft model using nude mice treated with daily oral enzalutamide (10 mg/kg). **N**, **O** Bioluminescence imaging and quantification revealed that circPPFIA2-overexpressing 22Rv1 xenografts exhibited attenuated response to enzalutamide, with sustained tumor signal intensity post-treatment. **P**–**R** Longitudinal tumor volume measurements and tumor weights measurement confirmed accelerated growth in circPPFIA2-overexpressing cohorts despite enzalutamide administration. **S** Histopathological analysis of xenograft tumors reveals circPPFIA2-overexpressing lesions retain high Ki-67 and ETS1 under enzalutamide treatment, correlating with therapeutic escape. Scale bars: 50 μm (H&E), 20 μm (IHC). Data are presented as mean ± SD (*n* = 3 biological replicates). Two-tailed tests with Bonferroni correction were used; *p < 0.05, **p < 0.01, ***p < 0.001, ****p < 0.0001.

To determine the key role of circPPFIA2 overexpression in the development of enzalutamide resistance in CRPC cells, we treated C4-2 cells with enzalutamide and found that decreasing circPPFIA2 levels effectively mitigated the resistance of PCa cells to enzalutamide (Fig. 6A), whereas increasing circPPFIA2 exert opposite effects (Fig. 6B). These effects were recapitulated in 22Rv1 and enzalutamide-resistant C4-2^ENZR^ cells (Fig. 6C, D, and Supplementary Fig. S6A, B), concomitant with elevated circPPFIA2 and ETS1 expression (Fig. 6E, F). Functional studies demonstrated that circPPFIA2 knockdown in resistant cells restored enzalutamide sensitivity, whereas its overexpression in sensitive cells conferred resistance, as confirmed by CCK-8 proliferation assays, colony formation and EdU staining analyses (Supplementary Fig. S6C–J). To further evaluate therapeutic delivery, we formulated lipid nanoparticles (LNPs) encapsulating si-circPPFIA2, enzalutamide, or both, and characterized their physicochemical properties. Co-delivery LNPs exhibited uniform nano-spheres with high co-encapsulation (si-circ EE% > 89%, enzalutamide EE% > 84%, Fig. 6G–J, and Supplementary Fig. S6K, L). Then qPCR demonstrated effective circPPFIA2 silencing in C4-2^ENZR^ and 22Rv1 cells following treatment with si-circ-LNP and ENZ/si-circ-LNP (Supplementary Fig. S6M). Co-delivery LNPs elicited circPPFIA2-mediated re-sensitization to enzalutamide, with superior antiproliferative effects compared to single-agent treatments, as supported by CCK-8, colony formation, and EdU results (Fig. 6K, L, and Supplementary Fig. S6N, O). In vivo, PSA-expressing xenografts overexpressing circPPFIA2 maintained robust tumor growth under enzalutamide treatment (10 mg/kg), displaying accelerated growth rates and increased tumor weights compared to regressing controls (Fig. 6M–R). Histopathological analysis confirmed treatment-refractory phenotypes characterized by sustained Ki-67 expression and upregulated ETS1 levels (Fig. 6S). These findings establish circPPFIA2 as a central mediator of enzalutamide resistance in PCa, highlighting its dual potential as a predictive biomarker and therapeutic target in advanced disease.

## Discussion

Circular RNAs (circRNAs), covalently closed RNA molecules derived from back-splicing of pre-mRNAs, have emerged as critical regulators of tumorigenesis through their roles in chromatin remodeling, protein interactions, and post-transcriptional modulation [22–24]. While dysregulated circRNAs have been implicated in diverse malignancies including PCa, their mechanistic contributions to therapy resistance and metastatic progression remain incompletely characterized [25–29]. In this study, we identify circPPFIA2 as a novel oncogenic circRNA that is significantly upregulated in PCa tissues and correlates with advanced clinical stages, accelerated biochemical recurrence, and reduced overall survival. Functional studies in vitro and in vivo demonstrate that circPPFIA2 drives tumor proliferation, metastatic dissemination, and enzalutamide resistance, positioning it as both a prognostic biomarker and therapeutic target.

Understanding circRNA biogenesis in PCa is critical for deciphering how specific circRNAs are produced and accumulate during disease progression, yet this area remains understudied. Multiple RNA-binding proteins (RBPs) are established regulators of circular RNA biogenesis, with roles ranging from promoting (e.g., QKI), modulating (e.g., FUS), or suppressing (e.g., DHX9) back-splicing [30–32]. Among them, HNRNPL has emerged as a context-dependent facilitator of circRNA formation, known to bind CA-rich motifs in flanking introns to promote back-splicing of certain transcripts [33]. Intriguingly, however, HNRNPL can also suppress circRNA production in other settings, as exemplified by its inhibition of circCANX biogenesis through enhanced linear splicing of CANX pre-mRNA—a process linked to p53 accumulation [34]. These findings highlight the gene-specific role of HNRNPL in circRNA regulation. In the present study, we provide direct experimental evidence that HNRNPL binds specific intronic regions of PPFIA2 pre-mRNA and promotes its back-splicing, thereby enhancing circPPFIA2 production. Collectively, our data support a model in which HNRNPL facilitates circPPFIA2 biogenesis through direct RNA binding and splicing regulation. We acknowledge that further mechanistic studies, such as locus-specific functional assays, will help establish definitive causality and clarify the contextual determinants of HNRNPL-mediated circRNA biogenesis.

CircRNAs exhibit diverse biological functions, with established roles as microRNA sponges that critically regulate cellular processes [35]. For instance, circNOLC1 serves as a diagnostic biomarker in PCa [36], while circBNC2 and circSLC4A7 promote chemoresistance through miRNA sponging mechanisms [37, 38]. Building on these paradigms of circRNA-mediated oncogenesis, our results demonstrate that circPPFIA2 predominantly localized in the cytoplasm, functions as a competitive endogenous RNA (ceRNA). Specifically, it sequesters miR-646 and miR-1200, thereby derepressing ETS1, a transcriptional effector that orchestrates core oncogenic signaling pathways. This mechanistic model is robustly supported by convergent experimental evidence. Firstly, RNA pull-down, Ago2 RIP and dual-luciferase assays confirmed direct physical interaction between circPPFIA2 and miR-646/miR-1200, with binding specificity abolished by mutagenesis of predicted response elements. Secondly, functional interrogation further revealed that miR-646/miR-1200 mimic delivery reversed circPPFIA2-driven proliferative and migratory phenotypes, while miRNA inhibition exacerbated tumor progression, underscoring their tumor-suppressive roles. Finally, circPPFIA2 bidirectionally regulated ETS1 expression, and rescue experiments demonstrated that ETS1 ablation fully abrogated oncogenic effects of circPPFIA2, establishing ETS1 as the indispensable downstream mediator of this regulatory axis. Together, these findings delineate a ceRNA-driven circuit wherein circPPFIA2 hijacks miR-646/miR-1200 to amplify ETS1-dependent survival and metastatic signaling, providing a molecular blueprint for therapeutic intervention for PCa.

Notably, miR-646 and miR-1200 have been previously characterized as tumor suppressors in genitourinary and other types of cancers [39–43], and our work extends their functional relevance to PCa. The identification of ETS1 as their downstream target is particularly significant, given the role of ETS1 in AR signaling, EMT, and therapy resistance [44, 45]. Clinical validation revealed elevated ETS1 levels in PCa tissues, inversely correlated with miR-646/miR-1200 expression and directly linked to circPPFIA2 upregulation. Strikingly, ETS1 knockdown abrogated circPPFIA2 induced proliferation and migration, while its overexpression mimicked the oncogenic effects of circPPFIA2, underscoring their functional synergy.

The translational implications of this axis are underscored by the role of circPPFIA2 in enzalutamide resistance. Specifically, we observed that overexpression of circPPFIA2 in CRPC xenograft models abrogated the therapeutic efficacy of enzalutamide, as evidenced by a lack of significant tumor regression following treatment, which establishes circPPFIA2 overexpression as a mechanism underlying treatment evasion, thus broadening the research horizon in the field of therapy resistance [46, 47]. Despite their tremendous therapeutic potential in cancer, the targeted delivery of circRNAs to tumor sites remains a significant challenge. Lipid nanoparticles enable targeted cancer therapy by encapsulating diverse cargoes including nucleic acids (siRNA, mRNA, shRNA, circRNA) and small-molecule drugs [48]. While delivery challenges persist, recent advances confirm their therapeutic feasibility, as exemplified by circRNA-targeted LNP systems suppressing tumor growth in prostate cancer models [49]. Building on this paradigm, we engineered a novel LNP platform encapsulating shRNA plasmids targeting circPPFIA2. This approach demonstrated robust anti-tumor efficacy in enzalutamide-resistant PCa models, advancing the translational potential of circRNA-directed nano-therapeutics.

It should be noted that our conclusions are derived from two representative yet limited models of resistance. The diverse mechanisms underlying resistance to AR-targeted therapies mean that findings from C4-2^ENZR^ and 22Rv1 cells alone may not be fully generalizable. Future work employing a wider array of independently derived resistant lines would be valuable to substantiate the role of circPPFIA2 across the broader therapeutic resistance landscape.

## Conclusion

**Fig. 7:**
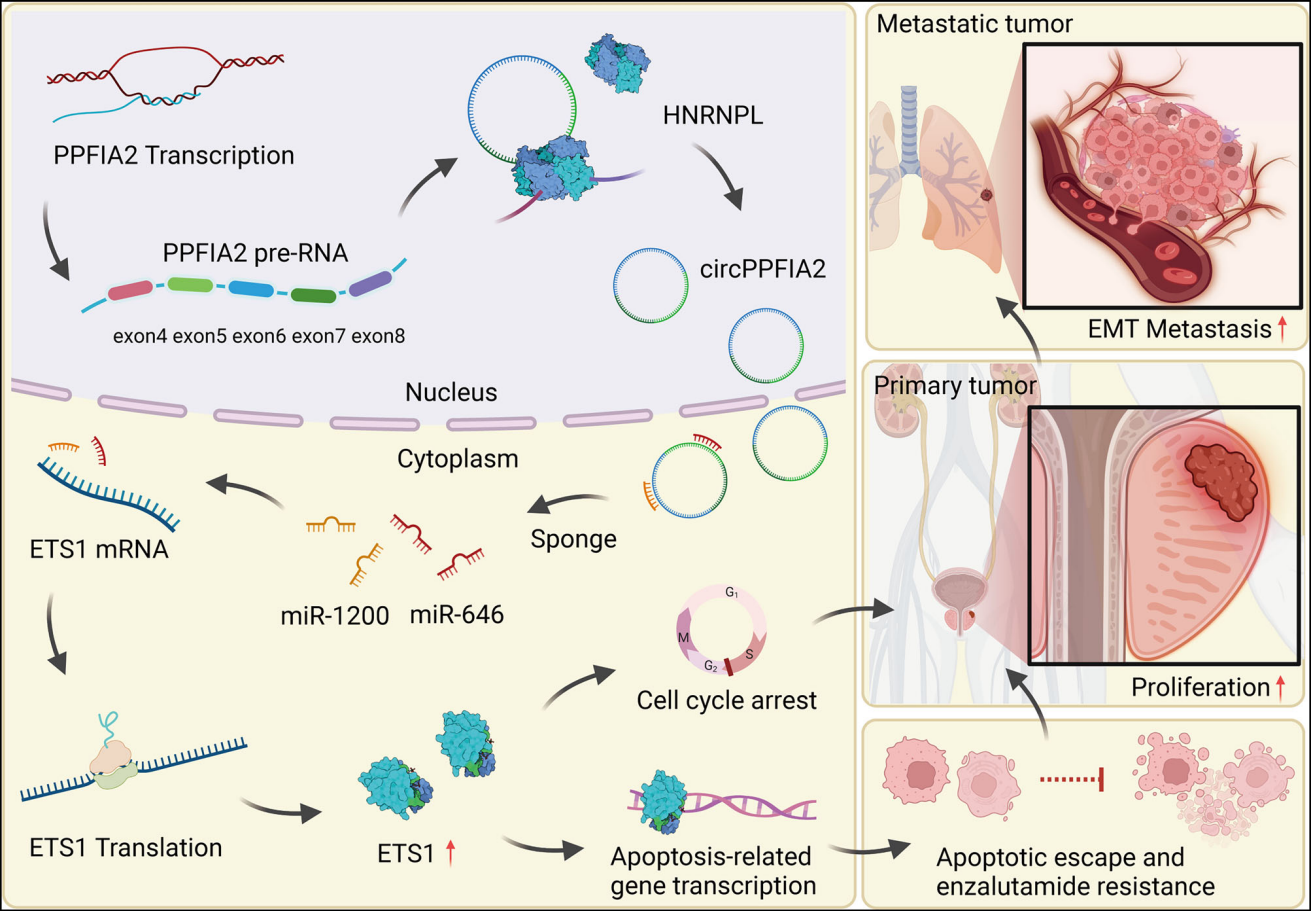
Mechanistic framework and oncogenic functions of circPPFIA2 in PCa pathogenesis. This schematic synthesizes preclinical evidence demonstrating the role of circPPFIA2 as a molecular sponge that competitively binds miR-646 and miR-1200, thereby derepressing ETS1, a transcriptional effector driving proliferative signaling, metastatic dissemination, and enzalutamide resistance in PCa. Created in BioRender. (Yiyou M. 2025 https://BioRender.com/i10o044).

Our study delineates the circPPFIA2/miR-646/miR-1200/ETS1 axis as a central driver of PCa progression and therapy resistance. By sponging tumor-suppressive miRNAs, circPPFIA2 unleashes ETS1-mediated transcriptional programs that fuel proliferation, metastasis, and enzalutamide resistance (Fig. 7). These findings highlight that targeting circPPFIA2 as a promising therapeutic target in PCa patients.

## Materials and Methods

### Patients and samples

A PCa tissue microarray (TMA) was procured from Outdo Biotech (Shanghai, China). Clinicopathological data, including age, Gleason score, survival status, and recurrence outcomes, are detailed in Supplementary Table S1.

### Cell culture

PCa cells (LNCaP, 22Rv1, DU145, PC3, C4-2) and normal prostate epithelial cells (RWPE-1) were obtained from Chinese Academy of Sciences Cell Bank. PCa cells were maintained in RPMI-1640 with 10% FBS. RWPE-1 cells were cultured in KSFM (Gibco #10744-019) supplemented with 5 ng/mL EGF (Gibco #10450-013) and 1% antibiotic-antimycotic (Gibco #15140-122). All cells incubated at 37 °C in 5% CO₂.

### Reverse transcriptase-polymerase chain reaction (PCR) and quantitative real-time PCR assays(qRT-PCR)

Total RNA was extracted using TRIzol reagent (Invitrogen, USA), followed by cDNA synthesis with 5×PrimeScript™ Master Mix (TaKaRa, China). qRT-PCR was performed using SYBR Premix Ex Taq II (Takara Bio, Japan) on a LightCycler 480 system (Roche, Switzerland). Each reaction contained 10 μL of SYBR Green mix, 0.4 μL each of forward and reverse primers (10 μM), 2 μL of cDNA template, and nuclease-free water to a final volume of 20 μL. The thermal cycling conditions were 95 °C for 30 s, followed by 40 cycles of 95 °C for 5 s and 60 °C for 30 s. Relative gene expression was calculated using the 2^–ΔΔCt^ method, with GAPDH as the internal control. All reactions were performed in triplicate. Primer sequences are listed in Supplementary Table S2.

### Gene modulation

siRNAs targeting circPPFIA2, HNRNPL, ETS1, and NC siRNA, along with miR-646/miR-1200 mimics/inhibitors (RiboBio), were transfected using Lipofectamine 3000 (Invitrogen #L3000015). Specific siRNA sequences are provided in Supplementary Table S2. Lentiviral vectors (GeneChem) generated stable circPPFIA2-knockdown or -overexpressing cell lines selected with 5 μg/mL puromycin for 10 days. Modulation efficiency was validated by qRT-PCR or Western blotting.

### Cell viability assay

Transfected PCa cells (1000 cells/well) in 96-well plates were monitored for 120 h using CCK-8 (Dojindo) at 24 h intervals. For enzalutamide sensitivity, circPPFIA2-modulated C4-2/22Rv1 cells (5000 cells/well) were treated with enzalutamide (Selleckchem #S1250; 0.1-100 μM) or 0.1% DMSO (72 h) before CCK-8 analysis. Experiments were performed in triplicate (*n* = 3) with independent biological samples. Dose-response curves and IC50 values were generated by four-parameter logistic regression (GraphPad Prism 9.0).

### Western blot analysis

Protein lysates were extracted using RIPA buffer (#KGP250, KeyGEN Bio) and quantified via BCA assay (#KGP902, KeyGEN Bio). Samples (30 μg) were resolved on 10% SDS-PAGE gels and transferred to PVDF membranes (#IPVH00010, Millipore). After blocking with 5% non-fat milk/TBST, membranes were incubated overnight at 4 °C with antibodies: anti-ETS1 (#ab307672, 1:1000, Abcam), EMT (#9782), Cell Cycle (#9932), and Apoptosis (#9915) Sampler Kits (1:1000, CST), diluted in Primary Antibody Buffer (#abs954, Absin). After TBST washes, HRP-conjugated anti-rabbit (#7074S) or anti-mouse (#7076S) secondary antibodies (1:3000, CST) were applied for 1 h. Bands were visualized using ECL reagent (#KF8001, Affinity Biosciences) and quantified via ImageJ (NIH).

### Dual-Luciferase Reporter Assay

Wild-type or mutant circPPFIA2 sequences were cloned into the pEZX-MT06 vector (GeneCopoeia, USA). The wild-type insert contained the predicted miR-1200 and miR-646 sites (bp 188–194 and 323–329), and the mutant construct carried seed-matching substitutions at these sites; mutations were introduced by site-directed mutagenesis during commercial synthesis and verified by Sanger sequencing. For ETS1, a 3’UTR fragment harboring the predicted miR-1200 and miR-646 sites (bp 2156–2162 and 2303–2309) was cloned into the same backbone, with a corresponding MRE-Mut reporter generated likewise. HEK-293T cells were co-transfected with reporter plasmids and miR-646/miR-1200 mimics (or negative control). At 48 h post-transfection, firefly and Renilla activities were measured using the Dual-Luciferase Reporter Assay System (Promega, USA); firefly was normalized to Renilla and expressed relative to miR-NC. Data represent three independent experiments.

### Biotin-labeled RNA pull-down

Biotinylated circPPFIA2 probes (RiboBio, China) were incubated with streptavidin-coated magnetic beads (Beaver Bio, China) and C4-2 cell lysates. RNA-protein complexes were isolated using the Pierce™ Magnetic RNA-Protein Pull-Down Kit (Thermo Fisher, USA), followed by qRT-PCR analysis.

### RNA Immunoprecipitation (RIP)

RIP assays were performed with the Magna RIP Kit (Bersinbio, China) following the manufacturer’s instructions. For the HNRNPL–circPPFIA2 assay, cell lysates were incubated with anti-HNRNPL (Abcam, ab6106) or species-matched normal IgG, and RNA recovered from immunocomplexes was analyzed by qRT-PCR. For the Ago2–circPPFIA2 assay, C4-2 cells stably expressing FLAG-Ago2 were generated by lentiviral transduction followed by puromycin selection; clarified lysates (~1–2 mg total protein per IP) were incubated with rabbit anti-FLAG (Proteintech, Cat. 20543-1-AP, 5 μg per IP) or normal rabbit IgG pre-bound to Protein G magnetic beads (~25 μL per IP) for 3 h at 4 °C with rotation. Beads were washed with high-salt RIP buffer (25 mM Tris-HCl pH 7.4, 300 mM KCl, 2 mM EDTA, 0.5% NP-40) and 1× with PBS; RNA was purified per the kit protocol (including DNase treatment). circPPFIA2 enrichment was quantified by qRT-PCR using primers spanning the back-splice junction and expressed as % input normalized to IgG. Data represent three independent biological replicates.

### RNA sequencing and data analysis

RNA sequencing was performed on C4-2/NC and C4-2/si-circPPFIA2 cells using the BGI-SEQ platform. Subsequent bioinformatic analyses included heatmap generation, Gene Set Enrichment Analysis (GSEA), and functional annotation via the Kyoto Encyclopedia of Genes and Genomes (KEGG) database.

### Immunohistochemistry (IHC)

Formalin-fixed paraffin-embedded sections (2.5 μm) underwent antigen retrieval and were incubated with primary antibodies against Ki-67 (ab15580, Abcam), ETS1 (ab307672, Abcam), Bcl2 (#15071, CST), and Bax (#5023, CST). HRP-conjugated secondary antibodies (Biyuntian, China) and DAB substrate (Zhongshan Jinqiao, China) were used for signal detection.

### Mouse Xenograft Models and drug administration

Animal protocols were approved by Zhujiang Hospital’s Animal Ethics Committee (LAEC-2024-201). Nude mice were randomly allocated to experimental groups using a random number table and then subcutaneously or intravenously injected with 22Rv1 cells (5×10^6^/mouse). Tumor growth/metastasis was monitored weekly via caliper measurements and bioluminescence imaging. From day 8 post-injection, enzalutamide (10 mg/kg) was administered intravenously every 5 days. Mice were euthanized on day 28 and tumors were harvested for IHC, FISH staining, and other histopathological analyses. For in vivo studies, each group included five mice (n = 5).

### Statistical analysis

Data are presented as mean ± SD. Group differences were assessed using Student’s t-test or one-way ANOVA followed by Bonferroni correction for multiple comparisons (GraphPad Prism 8). Survival analysis employed Kaplan–Meier curves with log-rank tests. *p* < 0.05 was considered statistically significant. n denotes biologically independent replicates; when technical replicates were present (e.g., qPCR wells), values were averaged within each biological replicate prior to statistical testing.

## Supplementary information

Supplementary Fig.

Supplementary Methods

Supplementary Table S1

Supplementary Table S2

Original Western Blot image

## Data Availability

All data that support the conclusions of this study are contained in the main text, figures, and the associated supplementary materials.
