# Supplementary material for: CircPPFIA2 drives prostate cancer progression and enzalutamide resistance by sponging miR-646 and miR-1200 to upregulate ETS1
Source: Cell Death Discov. 2025 Dec 8;12:45. doi: 10.1038/s41420-025-02904-z (PMC12830911; doi:10.1038/s41420-025-02904-z)
Supplement: Supplementary file 1 — Supplementary Fig. [file 41420_2025_2904_MOESM1_ESM.docx]

**SUPPLEMENTARY FIGURES**

**CircPPFIA2 Drives Prostate Cancer Progression and Enzalutamide Resistance by Sponging miR-646/miR-1200 to Upregulate ETS1**

Yiyou Mao^1,2†^, Qu Leng^2,5†^, Jun Wu^2†^, Wenbin Chen^6^, Chunxi Lin^8^, Zhihai Deng^2^, Qiang Shen^1^, Jun Zou^7^, Zining Long^2^, Yiyuan Zhan^2^, Shilong Cheng^2^, Zhongjie Chen^2^, Rui Zhou^2^, Jiaxing Wang^2^, Hangyang Peng^2^, Yangbai Lu^5^, Yilan Huang^9^, Chenglu Li^9^, Aihua Cai^9^, Jingyan Xu^9^, Hongxing Huang^5✉^, Dongmei Jiang^4✉^, Xiangming Mao^2✉^, Daojun Lv^1,3✉^

1 Department of Urology, Guangdong Provincial Key Laboratory of Major Obstetric Diseases, Guangdong Provincial Clinical Research Center for Obstetrics and Gynecology, The Third Affiliated Hospital of Guangzhou Medical University, Guangzhou, 510150, China.

2 Department of Urology, Zhujiang Hospital, Southern Medical University, Guangzhou, 510280, China.

3 Guangdong Provincial Key Laboratory of Urological Diseases, Guangzhou Medical University, Guangzhou, 510275, China.

4 Department of Pathology, The First Affiliated Hospital, Guangzhou Medical University, Guangzhou, 510120, China.

5 Department of Urology, Zhongshan City People's Hospital, Zhongshan, Guangdong, 528403, China.

6 Department of Urology, Shanghai Ninth People’s Hospital, Shanghai Jiao Tong University School of Medicine, Shanghai, 200011, China

7 Department of Emergency, Guangdong Provincial Key Laboratory of Major Obstetric Diseases, Guangdong Provincial Clinical Research Center for Obstetrics and Gynecology, The Third Affiliated Hospital, Guangzhou Medical University, Guangzhou, 510150, China.

8 The Third Clinical College, Guangzhou Medical University, Guangzhou, 511495, China

9 The Second Clinical College, Guangzhou Medical University, Guangzhou, 511495, China
† These authors have contributed equally to this work.

✉ Corresponding author: [daojunlv88@gzhmu.edu.cn](mailto:daojunlv88@gzhmu.edu.cn)

**This file includes:**

Supplementary Figure 1: HNRNPL Downregulation Enhances circPPFIA2 Biogenesis in Prostate Cancer.

Supplementary Figure 2: CircPPFIA2 Drives Prostate Cancer Proliferation and Migration via Cell Cycle and Apoptosis Regulation.

Supplementary Figure 3: CircPPFIA2 Drives Oncogenic Phenotypes via miR-646/miR-1200 Sponging.

Supplementary Figure 4: CircPPFIA2 Upregulates ETS1 via Dual miRNA Sponging to Activate Oncogenic Pathways.

Supplementary Figure 5: circPPFIA2 Drives PCa Progression via the miR-646/miR-1200/ETS1 Axis.

Supplementary Figure 6: CircPPFIA2 Modulates Enzalutamide Sensitivity in Prostate Cancer via ETS1 Signaling.


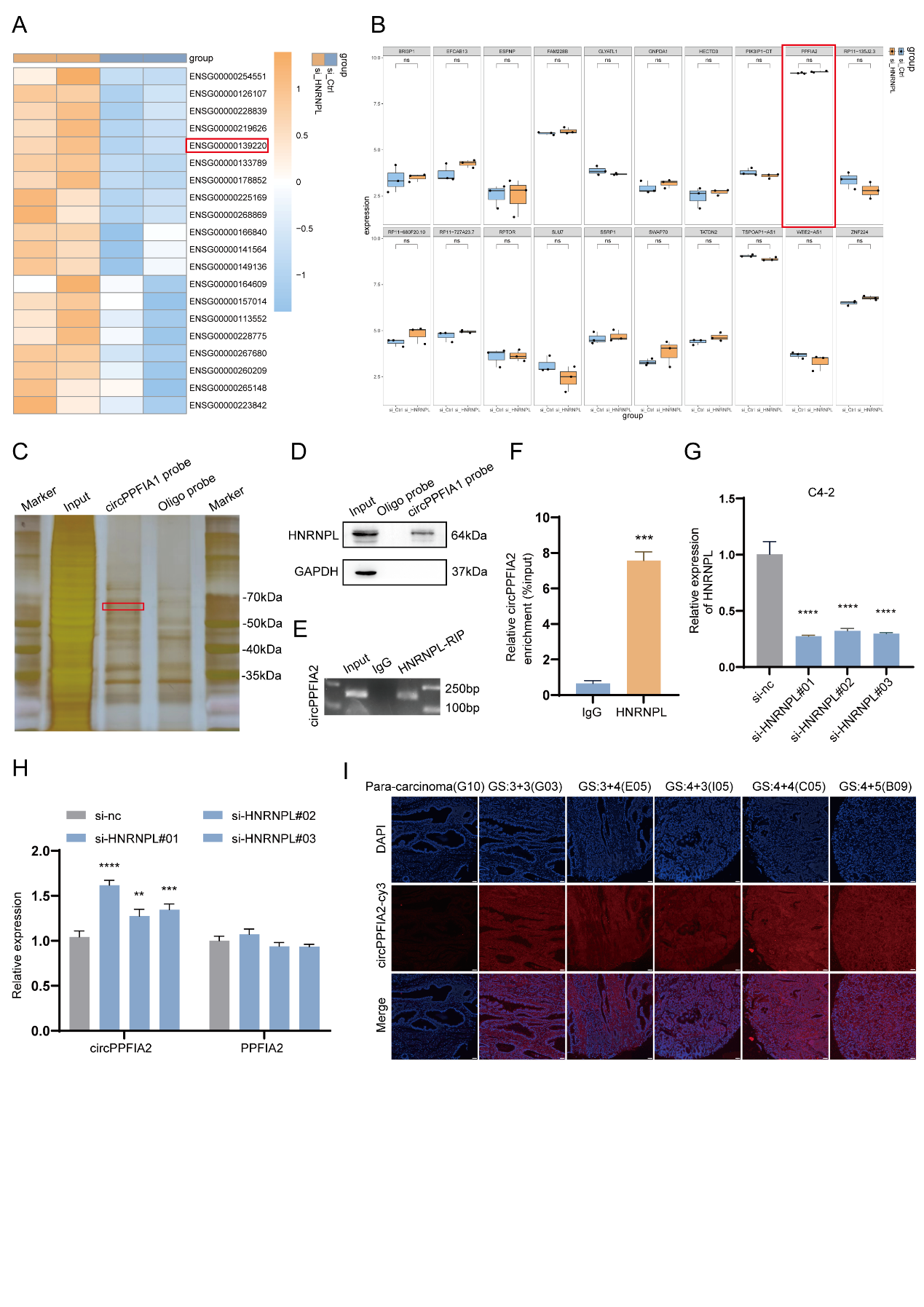


**Fig. S1. HNRNPL Downregulation Enhances circPPFIA2 Biogenesis in Prostate Cancer**
**(A)** Heatmap of differentially expressed circRNAs (top 20 upregulated/downregulated) in LNCaP cells after HNRNPL knockdown (si_HNRNPL vs. si_Ctrl; *p* < 0.05, |log2FC| > 2). **(B)** Box plot confirms unaltered linear *PPFIA2* mRNA levels in the transcriptome. **(C-D)** RNA pull-down with biotinylated circPPFIA2 probes followed by silver staining and Western blot identifies HNRNPL as a direct interactor. **(E-F)** RIP assay using anti-HNRNPL antibody validates circPPFIA2 enrichment (vs. IgG control). **(G)** qRT-PCR verifies HNRNPL knockdown efficiency (siRNAs #1–3**). (H)**HNRNPL silencing selectively elevates circPPFIA2 without affecting linear *PPFIA2* mRNA. (**I**) RNA-FISH analysis revealed pronounced cytoplasmic overexpression of circPPFIA2 in prostate cancer (PCa) tissues, showing significant positive correlation with advanced Gleason scores. Scale bar: 50 μm. Data: mean ± SD; ***p* < 0.01, ****p* < 0.001, *****p* < 0.0001.


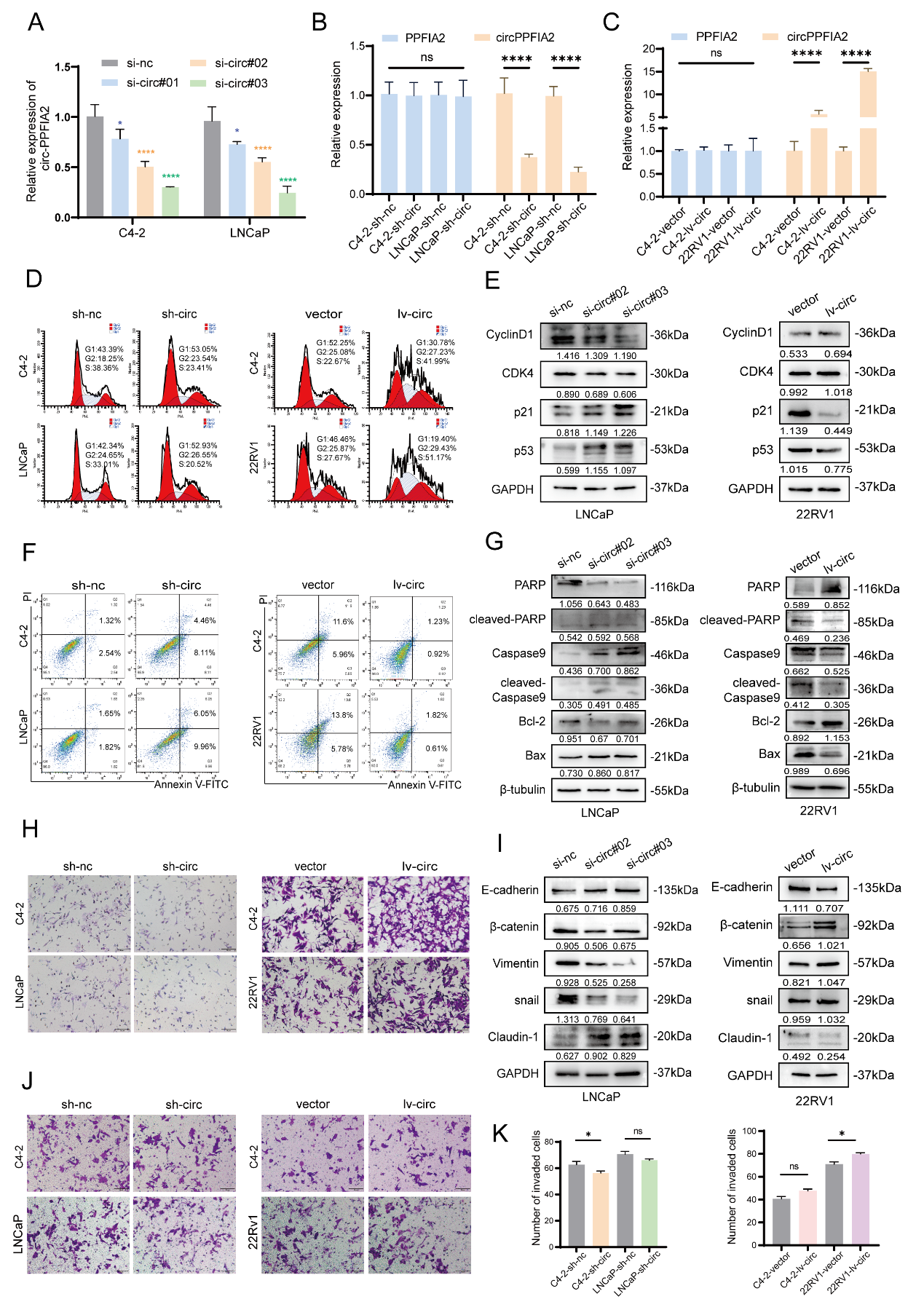


**Fig. S2.** **CircPPFIA2 Drives Prostate Cancer Proliferation and Migration via Cell Cycle and Apoptosis Regulation. (A)** Validation knockdown efficiency of three sequence-specific siRNAs (si-circ#01/02/03) targeting circPPFIA2 through qRT‑PCR. **(B-C)** Lentiviral-mediated circPPFIA2 knockdown or overexpression selectively modulates circular isoform levels without altering linear *PPFIA2* mRNA.**(D-E)** circPPFIA2 silencing induces G1/S phase arrest *via* Cyclin D1/CDK4 downregulation and p53/p21 activation, while overexpression accelerates cell cycle progression.**(F-G)**Apoptosis assays reveal circPPFIA2 knockdown increases apoptotic rate with elevated cleaved PARP and Bax/Bcl-2 ratio, reversed by overexpression. **(H-I)**Transwell migration assays demonstrate circPPFIA2 knockdown reduces motility, accompanied by E-cadherin restoration and vimentin suppression, whereas overexpression enhances metastatic potential. **(J-K)**Matrigel-coated Transwell invasion with circPPFIA2 perturbation in C4-2, LNCaP, and 22Rv1; images (J) and quantification (K). Differences were modest and not uniformly significant (n = 3; two-tailed tests; Bonferroni-adjusted P). Data: mean ± SD; ns, not significant, **p* < 0.05, *****p* < 0.0001.


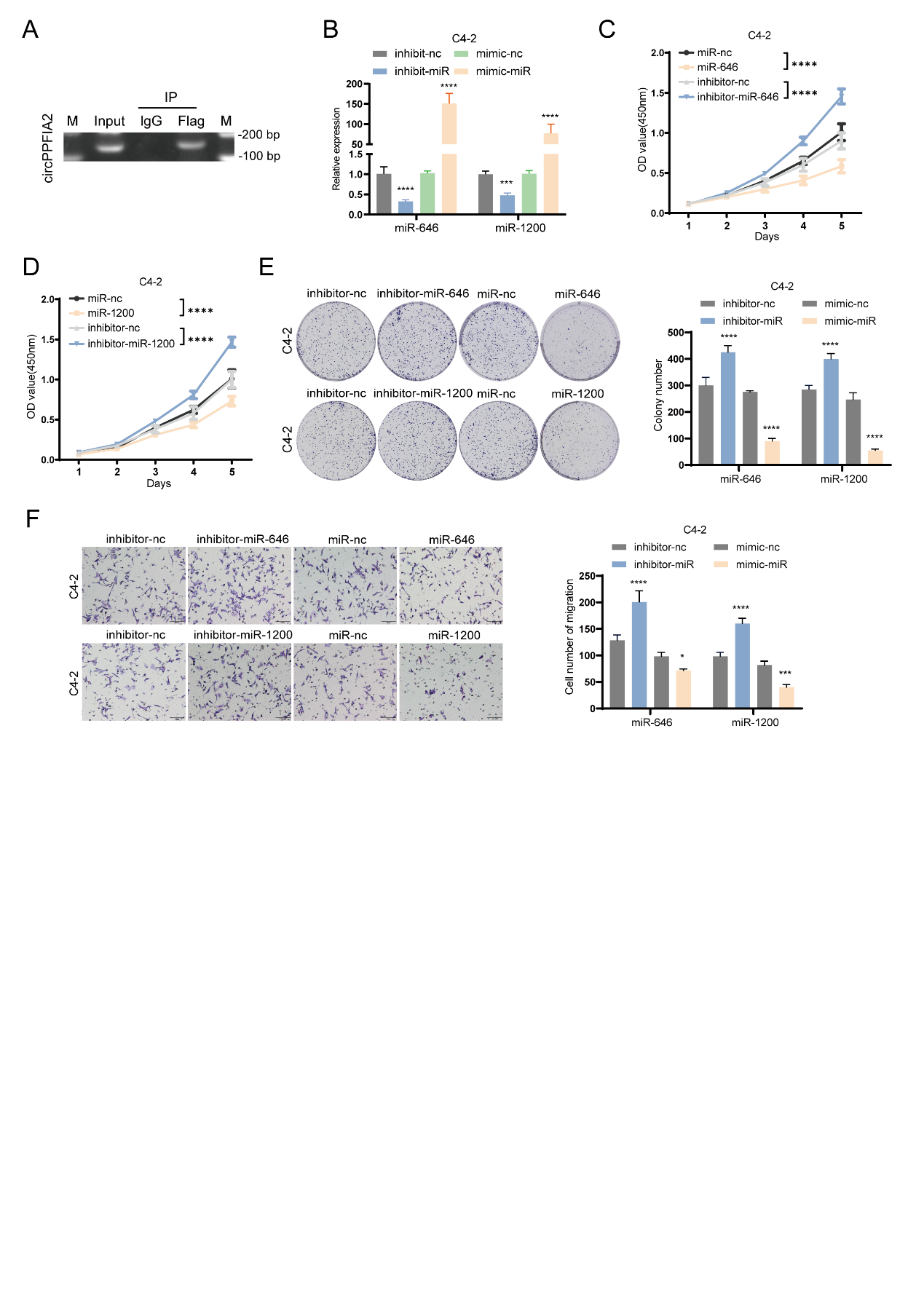


**Fig. S3.** **CircPPFIA2 Drives Oncogenic Phenotypes *via* miR-646/miR-1200 Sponging.** **(A)** Ago2 RIP—agarose gel of circPPFIA2 RT-PCR. The junction-spanning amplicon is present in input and FLAG-Ago2 IP but negligible in IgG; M, DNA ladder. Representative of three biological replicates. **(B)** qRT-PCR validates efficient miR-646/miR-1200 overexpression or silencing. **(C-F)** Functional assays demonstrate miR-646/miR-1200 knockdown rescues circPPFIA2-driven oncogenicity, while miRNA mimics suppress circPPFIA2-overexpression effects. Data: mean ± SD; ***p* < 0.01, ****p* < 0.001.


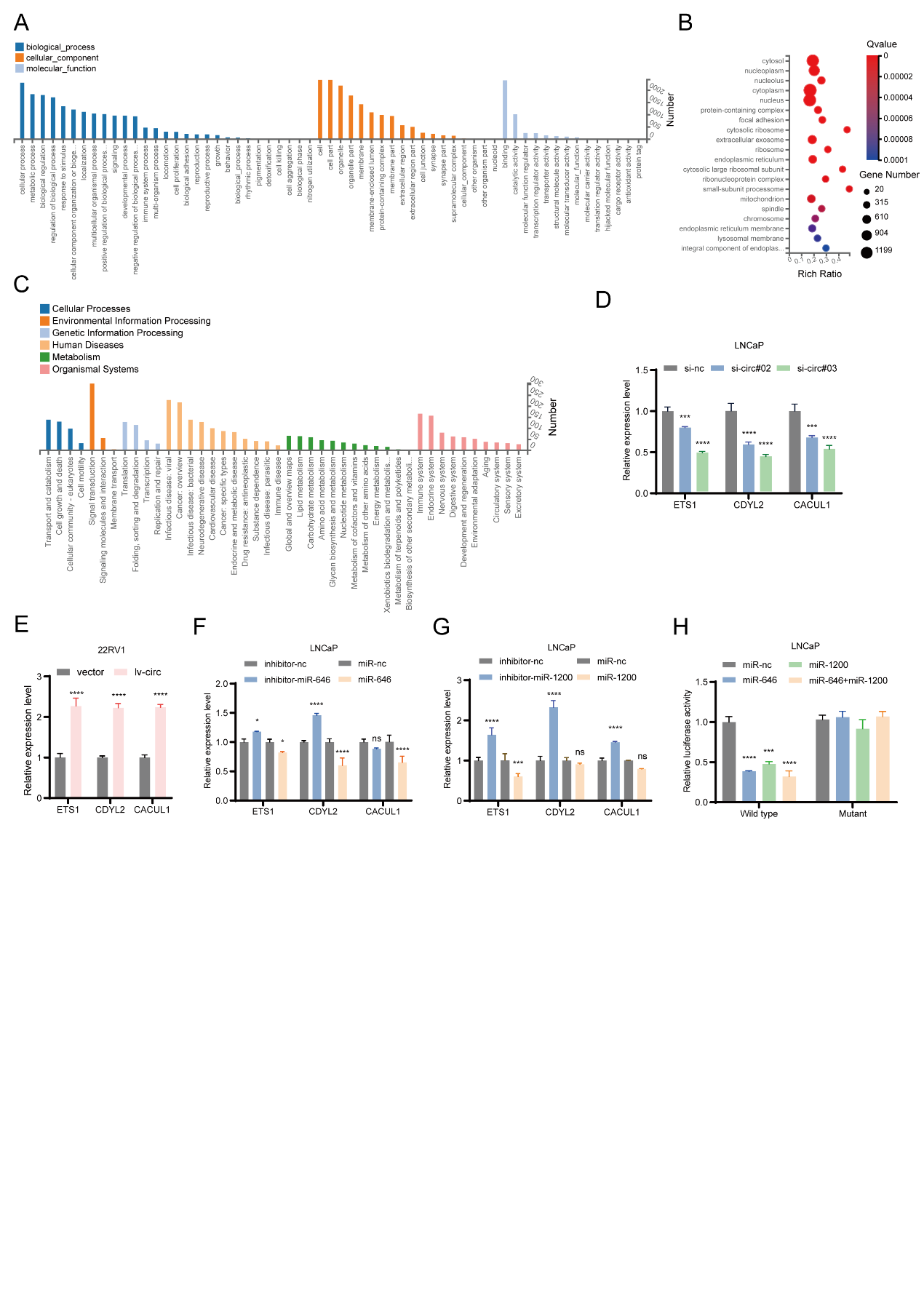


**Fig. S4.** **CircPPFIA2 Upregulates ETS1 via Dual miRNA Sponging to Activate Oncogenic Pathways. (A-C)** KEGG and GO enrichment analyses of circPPFIA2-silenced C4-2 cells identify apoptosis and p53 signaling as top dysregulated pathways. **(D-E)** circPPFIA2 knockdown reduces *ETS1* mRNA in LNCaP cells, while overexpression elevates *ETS1* in 22RV1 cells. **(F-G)** miR-646/miR-1200 mimics suppress *ETS1* expression, whereas inhibitors restore *ETS1* levels in circPPFIA2-modulated models. **(H)** Dual-luciferase assays confirm direct targeting of *ETS1* 3'UTR by miR-646/miR-1200, with co-transfection reducing activity, abolished by MRE mutagenesis. Data: mean ± SD; **p* < 0.05, ****p* < 0.001, *****p* < 0.0001.


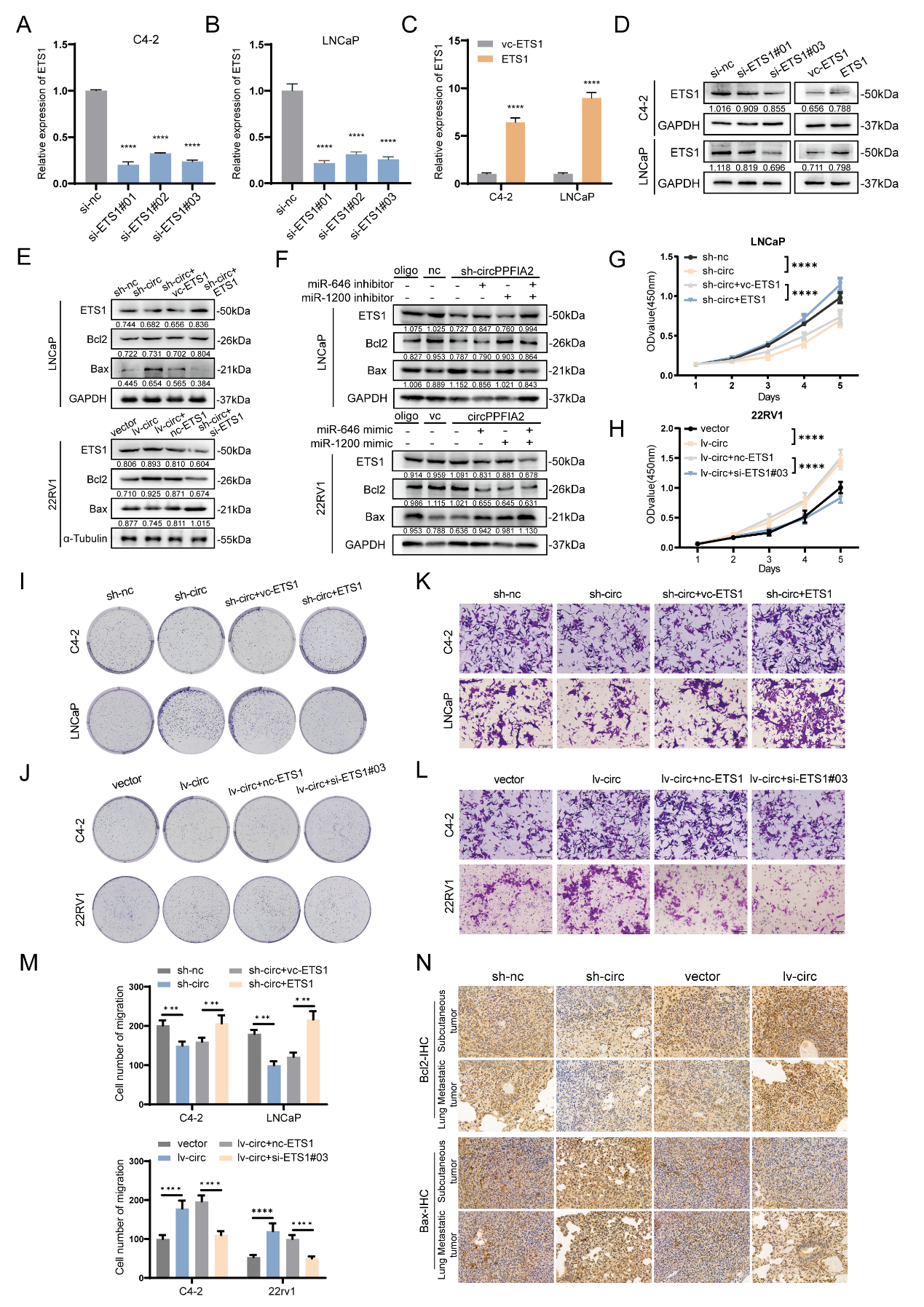


**Fig. S5.** **CircPPFIA2 Drives PCa Progression via the miR-646/miR-1200/ETS1 Axis**. **(A)** qRT-PCR validates efficient knockdown of *ETS1* by three independent siRNAs (si-ETS1#01/02/03) in C4-2 and LNCaP cells**. (B-C)***ETS1* overexpression plasmid significantly elevates mRNA levels in both cell lines. **(D)** Western blot confirms *ETS1* protein downregulation by siRNAs and upregulation by plasmid transfection. **(E)** Rescue experiments shows that *ETS1* reconstitution in circPPFIA2-silenced LNCaP cells restores Bcl2 (anti-apoptotic) and suppresses Bax (pro-apoptotic), while *ETS1* knockdown in circPPFIA2-overexpressing 22RV1 cells reverses these effects. **(F)** miR-646/miR-1200 inhibition in circPPFIA2-silenced cells restores *ETS1*/Bcl2, whereas miRNA mimics attenuate *ETS1*/Bcl2 in circPPFIA2-overexpressing cells. **(G-M)** Rescue experiments were performed to delineate the functional necessity of ETS1 in circPPFIA2-driven oncogenicity. CCK-8 and Transwell assays demonstrated that ETS1 overexpression in circPPFIA2-silenced cells restored migratory capacity and proliferative potential, while ETS1 knockdown in circPPFIA2-overexpressing models reversed these phenotypes. Scale bar: 50 μm. **(N)** IHC confirms circPPFIA2-driven dysregulation of apoptosis proteins (Bcl-2 and Bax) in subcutaneous tumors and lung metastases. Scale bar: 20 μm. Data: mean ± SD; *****p* < 0.0001.


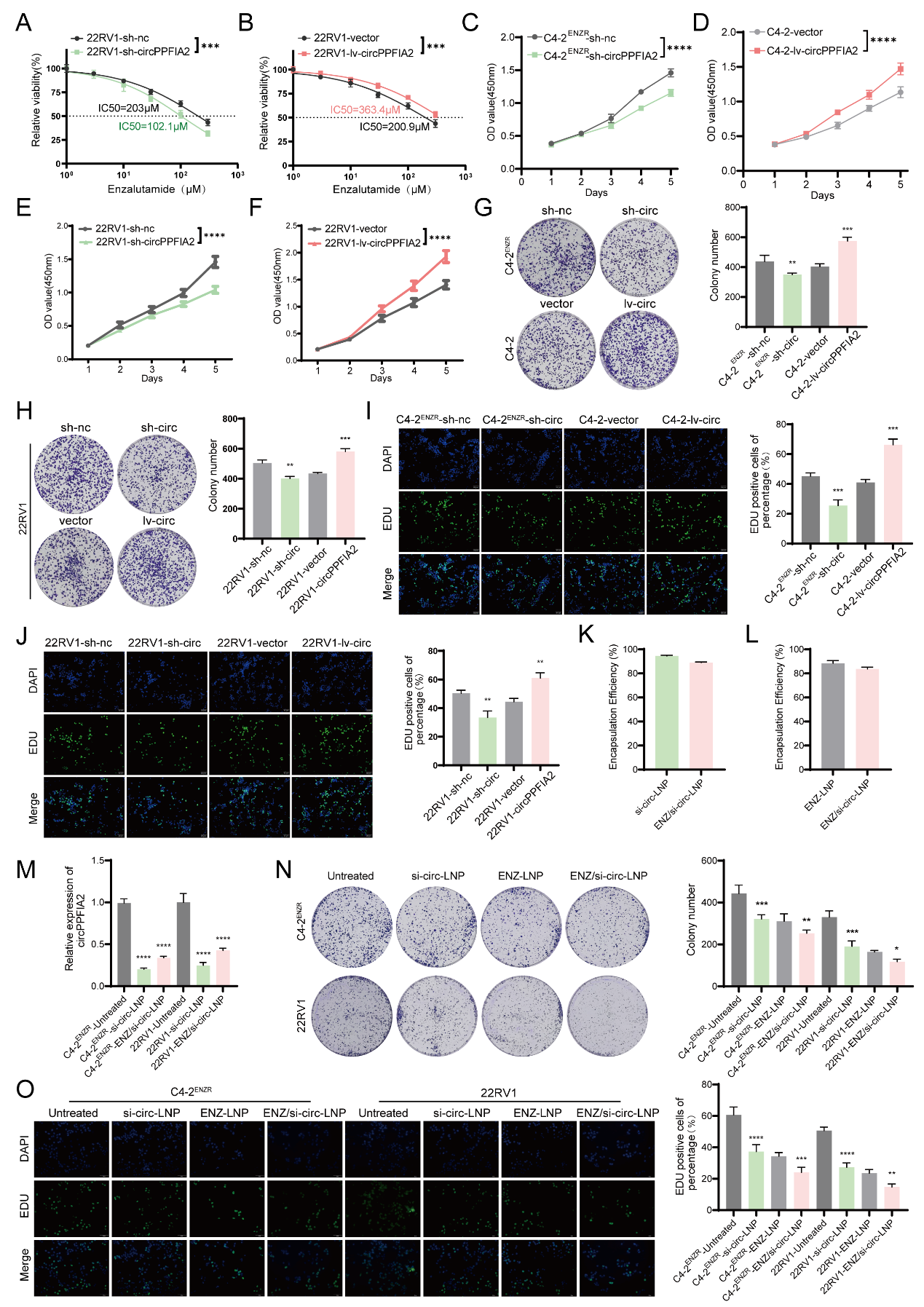


**Fig. S6. CircPPFIA2 Modulates Enzalutamide Sensitivity in Prostate Cancer via ETS1 Signaling. (A-B)** Consistent chemoresistance regulation was observed in 22RV1 cells, where circPPFIA2 silencing reduced enzalutamide IC50, whereas circPPFIA2 overexpression increased IC50. **(C-J)** Functional validation of circPPFIA2-driven therapy resistance: Knockdown of circPPFIA2 in 22RV1 or enzalutamide-resistant C4-2 cells suppressed proliferation under enzalutamide pressure (50.5 or 24.5 μM enzalutamide) in CCK-8, colony formation, and EdU assays, while circPPFIA2 overexpression in 22RV1 and parental C4-2 cells (50.5 or 58.25 μM enzalutamide) enhanced proliferative capacity. **(K-L)** Encapsulation efficiencies (EE) of si-circ (K) and enzalutamide (L) in single-agent and co-delivery LNPs, respectively. **(M)** qPCR analysis of circPPFIA2 expression in C4-2^ENZR^ and 22RV1 cells treated with si-circ-LNP or ENZ/si-circ-LNP. **(N-O)** Functional assays in C4-2ENZR and 22Rv1 cells after treatment with the indicated LNPs, including colony formation (O), and EdU incorporation (P) assays. Scale bars: 50 μm. Data: mean ± SD; *p < 0.05, **p < 0.01, ****p* < 0.001, *****p* < 0.0001.
