# Supplementary material for: CircPPFIA2 drives prostate cancer progression and enzalutamide resistance by sponging miR-646 and miR-1200 to upregulate ETS1
Source: Cell Death Discov. 2025 Dec 8;12:45. doi: 10.1038/s41420-025-02904-z (PMC12830911; doi:10.1038/s41420-025-02904-z)
Supplement: Supplementary file 2 — Supplementary Methods [file 41420_2025_2904_MOESM2_ESM.docx]

**Supplementary Methods**

**CircPPFIA2 Drives Prostate Cancer Progression and Enzalutamide Resistance by Sponging miR-646/miR-1200 to Upregulate ETS1**

Yiyou Mao^1,2†^, Qu Leng^2,5†^, Jun Wu^2†^, Wenbin Chen^6^, Chunxi Lin^8^, Zhihai Deng^2^, Qiang Shen^1^, Jun Zou^7^, Zining Long^2^, Yiyuan Zhan^2^, Shilong Cheng^2^, Zhongjie Chen^2^, Rui Zhou^2^, Jiaxing Wang^2^, Hangyang Peng^2^, Yangbai Lu^5^, Yilan Huang^9^, Chenglu Li^9^, Aihua Cai^9^, Jingyan Xu^9^, Hongxing Huang^5✉^, Dongmei Jiang^4✉^, Xiangming Mao^2✉^, Daojun Lv^1,3✉^

1 Department of Urology, Guangdong Provincial Key Laboratory of Major Obstetric Diseases, Guangdong Provincial Clinical Research Center for Obstetrics and Gynecology, The Third Affiliated Hospital of Guangzhou Medical University, Guangzhou, 510150, China.

2 Department of Urology, Zhujiang Hospital, Southern Medical University, Guangzhou, 510280, China.

3 Guangdong Provincial Key Laboratory of Urological Diseases, Guangzhou Medical University, Guangzhou, 510275, China.

4 Department of Pathology, The First Affiliated Hospital, Guangzhou Medical University, Guangzhou, 510120, China.

5 Department of Urology, Zhongshan City People's Hospital, Zhongshan, Guangdong, 528403, China.

6 Department of Urology, Shanghai Ninth People’s Hospital, Shanghai Jiao Tong University School of Medicine, Shanghai, 200011, China

7 Department of Emergency, Guangdong Provincial Key Laboratory of Major Obstetric Diseases, Guangdong Provincial Clinical Research Center for Obstetrics and Gynecology, The Third Affiliated Hospital, Guangzhou Medical University, Guangzhou, 510150, China.

8 The Third Clinical College, Guangzhou Medical University, Guangzhou, 511495, China

9 The Second Clinical College, Guangzhou Medical University, Guangzhou, 511495, China

† These authors have contributed equally to this work.

✉ Corresponding author: [daojunlv88@gzhmu.edu.cn](mailto:daojunlv88@gzhmu.edu.cn)

***Fluorescence In Situ Hybridization (FISH)***

Cy3-labeled circPPFIA2 probes and Alexa Fluor 488-labeled miR-646/miR-1200 probes (RiboBio, China) were hybridized to cells or tissues using the FISH Kit (C10910, RiboBio). Nuclei were counterstained with DAPI.

***RNase R and Actinomycin D Assays***

Total RNA (2 μg) was treated with/without RNase R (3 U/μg; Geneseed) at 37°C for 20 min. RNA was then purified (RNeasy MinElute Kit, Qiagen). For transcriptional inhibition, C4-2 and LNCaP cells were exposed to Actinomycin D (Sigma-Aldrich) for 4-24 h, with DMSO as control.

***Nuclear-cytoplasmic fractionation and RNA Isolation***

Nuclear-cytoplasmic fractionation was performed using the PARIS™ Kit (Invitrogen). Briefly, cells (1-2×10⁷) were washed with PBS, resuspended in 500 μL ice-cold cell separation buffer (10 min), and centrifuged at 500×g for 5 min. Supernatant (cytoplasm) and pellet (nucleus) were separated. Nuclear pellets were lysed in ice-cold lysis buffer. RNA isolation proceeded by adding equal volumes of 2×lysis/binding solution and ethanol to lysates, transferring to filter cartridges, and eluting with 50 μL pre-heated (95°C) elution solution. Fraction purity was verified by qRT-PCR using U6 (nuclear) and GAPDH (cytoplasmic) markers.

***Nucleic acid gel electrophoresis***

Nucleic acids with loading buffer were electrophoresed on 2% agarose gels containing ethidium bromide. Separation by size was achieved under electric field. DNA bands were visualized by UV illumination for analysis.

***Colony Formation Assay***

Cells were plated in 6-well plates (800 cells/well) and cultured in complete RPMI-1640 medium (Gibco) for 14 days. Colonies were fixed with 4% paraformaldehyde (PFA, Sigma-Aldrich) for 15 min, stained with 0.5% crystal violet (Baso Diagnostics, Zhuhai, China) for 10 min, and quantified using ImageJ software (v1.53). Colonies containing >50 cells were counted.

***5-ethynyl-2’-deoxyuridine (EdU) DNA Synthesis Assay***

Transfected cells in 96-well plates were pulsed with 50 μM EdU (Cell-Light™ EdU Kit, RiboBio, Guangzhou, China) for 2 hours after 48-hour culture. Cells were fixed with 4% PFA (20 min), permeabilized with 0.5% Triton X-100 (10 min), and stained with Apollo® fluorescent azide (30 min, dark). Nuclei were counterstained with DAPI (5 μg/mL, 15 min). EdU-positive cells were imaged at 200× magnification using an FSX100 microscope (Olympus, Tokyo, Japan), with ≥5 fields analyzed per well.

***Transwell migration assays***

Cell migration was assessed by using 8.0 μm pore polycarbonate membranes (Costar, Corning) according to the manufacturer’s instructions. Briefly, Cells were seeded in upper chambers for 24 h. After removing non-migrating cells, membranes were fixed with 4% PFA and stained with 0.1% crystal violet. Migrated cells were counted in ≥5 fields per membrane using ImageJ. Three independent experiments performed.

***Cell cycle analysis***

Cell cycle analysis was conducted following the manufacturer’s protocol. Briefly, cells were trypsinized, fixed in 70% ethanol (4°C overnight), stained with 50 μg/mL PI (Keygentec), and analyzed by flow cytometry (BD FACS Calibur).

***Cell apoptosis analysis***

Apoptosis was analyzed by flow cytometry (BD Biosciences) after 48-h treatments. Cells were stained with APC-Annexin V and PI (Vazyme), with quantification following manufacturer protocols.

***Preparation and Characterization of Lipid Nanoparticles (LNPs)***

Lipid nanoparticles (LNPs) encapsulating si-circPPFIA2 (si-circ) and/or enzalutamide were synthesized using a microfluidic mixing system (LNP-S1, Ponsan Biotech). For single-agent formulations, either si-circ (4.5 μg/mL) or enzalutamide (320 μg/mL) was dissolved in 50 mM sodium citrate buffer (pH 4.0) to form the aqueous phase. For co-delivery LNPs, both agents were co-dissolved in the same buffer. Each aqueous phase was mixed with an ethanol phase containing a commercial lipid formulation (FluidicLab LNP Kit) at a 3:1 aqueous-to-ethanol volumetric ratio and a total flow rate of 12 mL/min.

The resulting LNPs were diluted, purified by ultrafiltration (100 kDa MWCO), sterilized through a 0.22-μm filter, and stored at 4°C. Particle size, polydispersity index (PDI), and zeta potential were determined by dynamic light scattering, while morphology was assessed using transmission electron microscopy (TEM). Encapsulation efficiencies (EE%) were quantified via HPLC for enzalutamide and a fluorescence-based assay for si-circ. For co-delivery LNPs, EE% exceeded 84% for both agents. All LNP formulations were freshly prepared and characterized within 24 hours prior to use in downstream assays.
