# Supplementary material for: CircPPFIA2 drives prostate cancer progression and enzalutamide resistance by sponging miR-646 and miR-1200 to upregulate ETS1
Source: Cell Death Discov. 2025 Dec 8;12:45. doi: 10.1038/s41420-025-02904-z (PMC12830911; doi:10.1038/s41420-025-02904-z)

F2-H

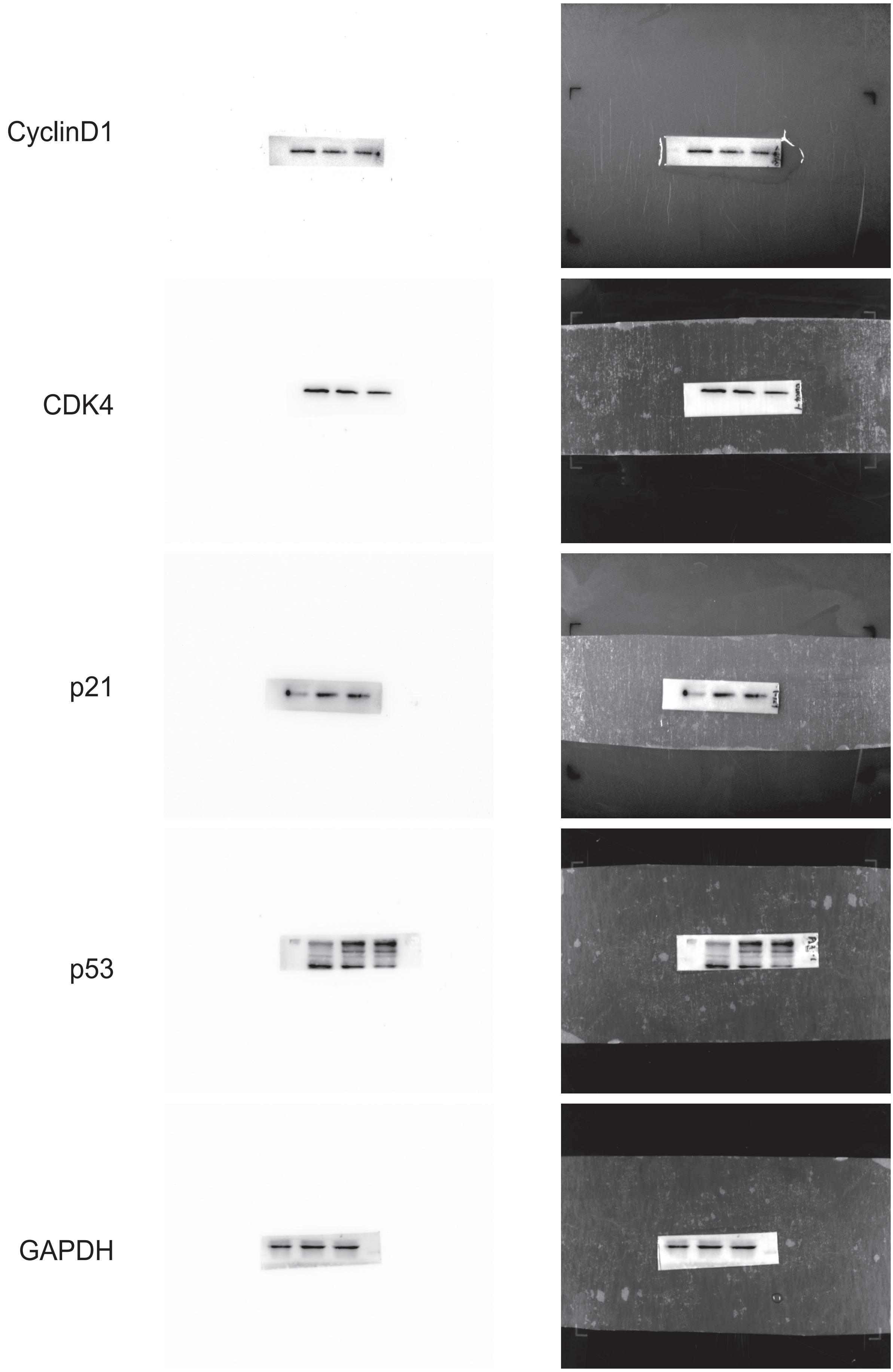

F2-J

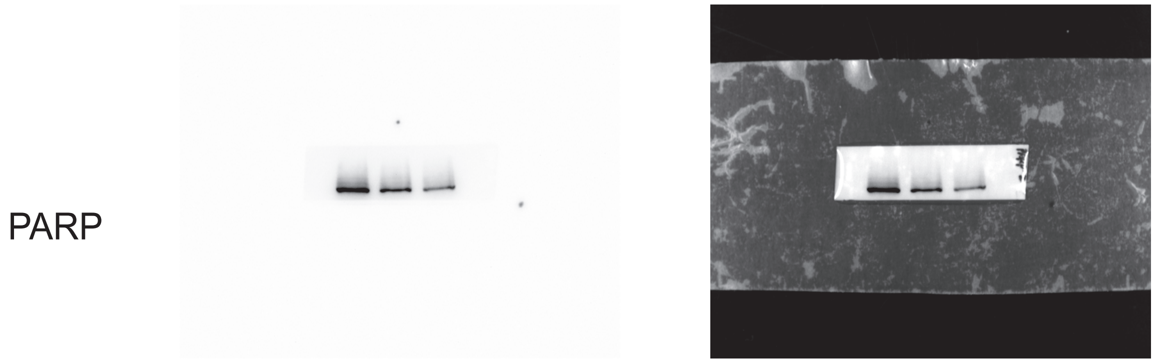

cleaved-PARP

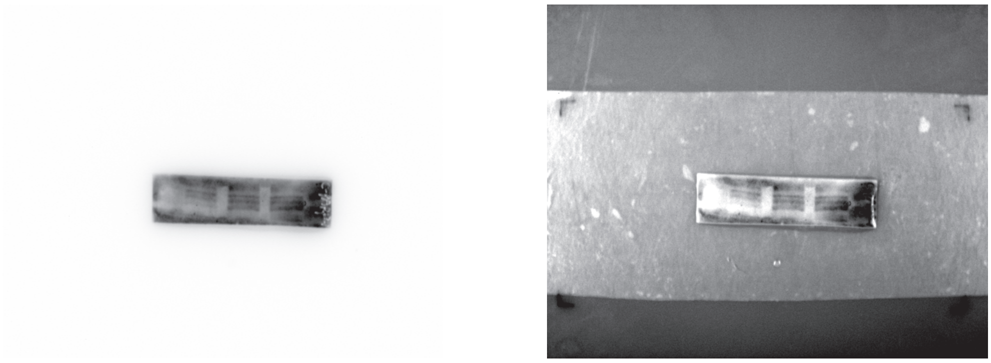

Caspase9

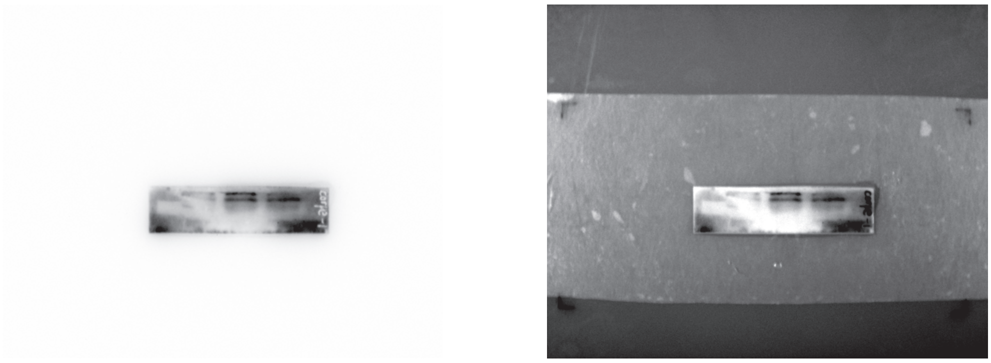

cleaved-Caspase9

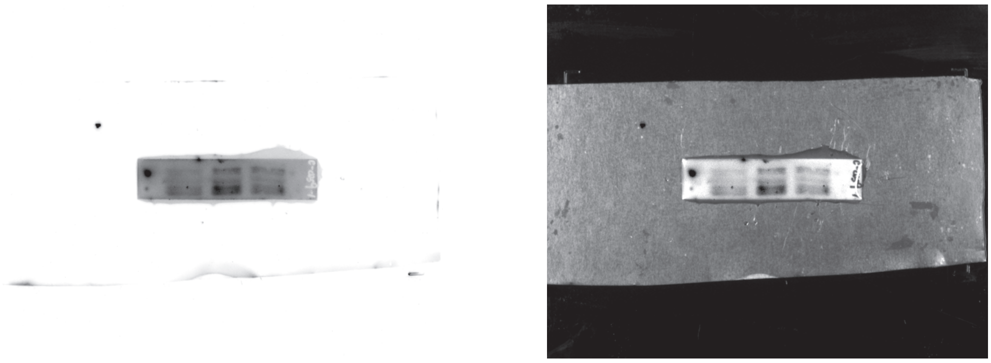

Bcl-2

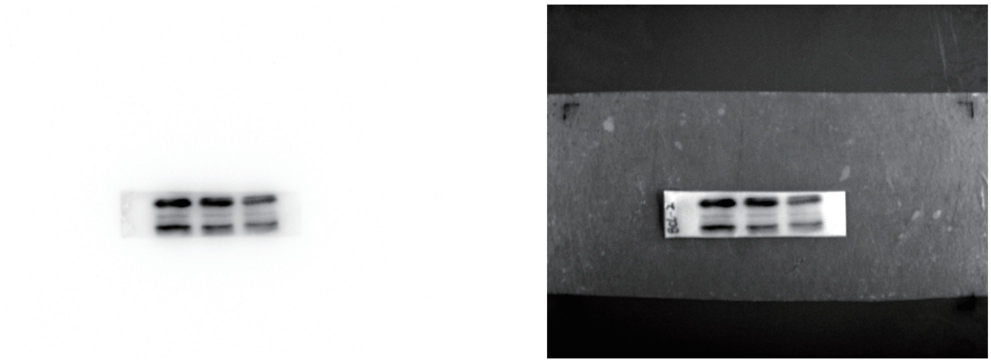

Bax

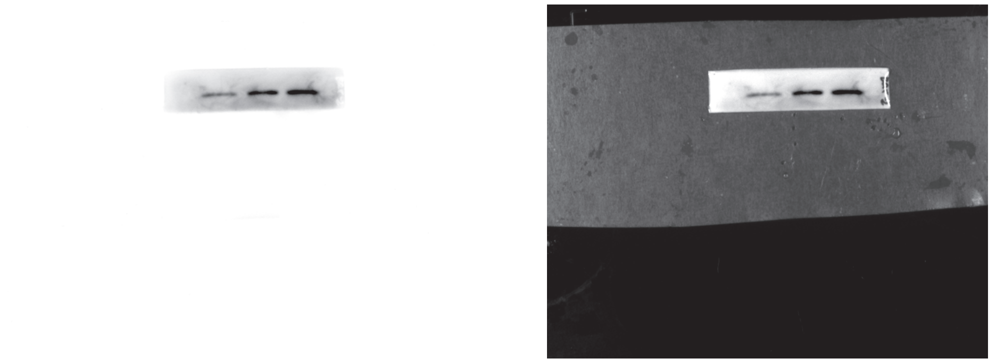

β-tubulin

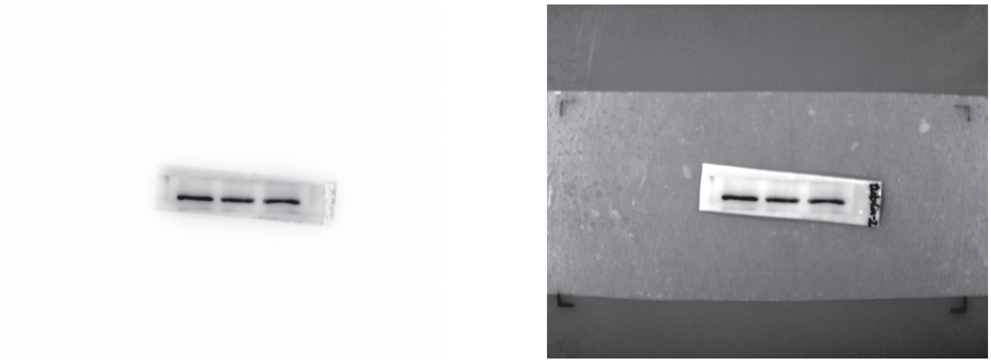

F2-H

CyclinD1

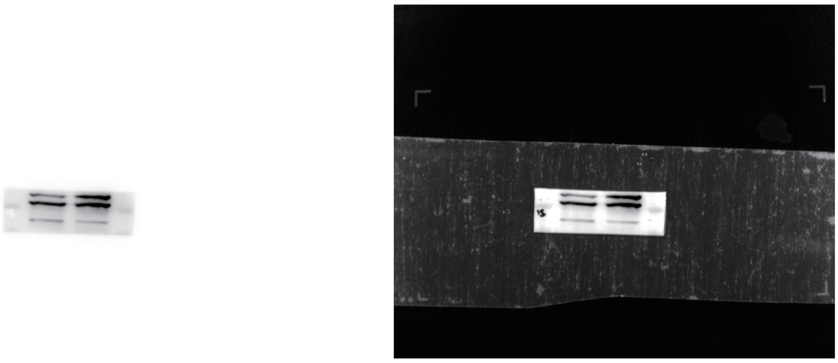

CDK4

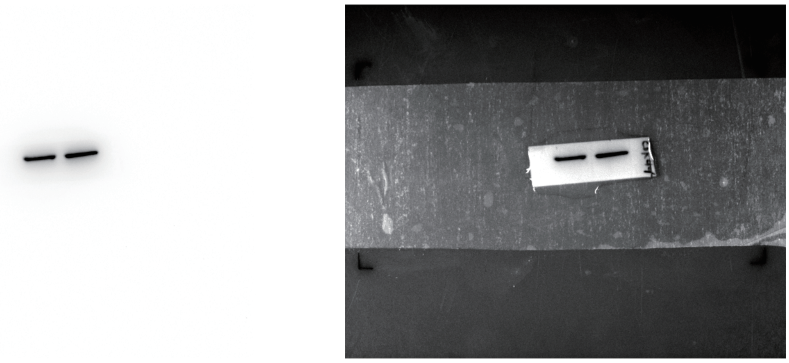

p21

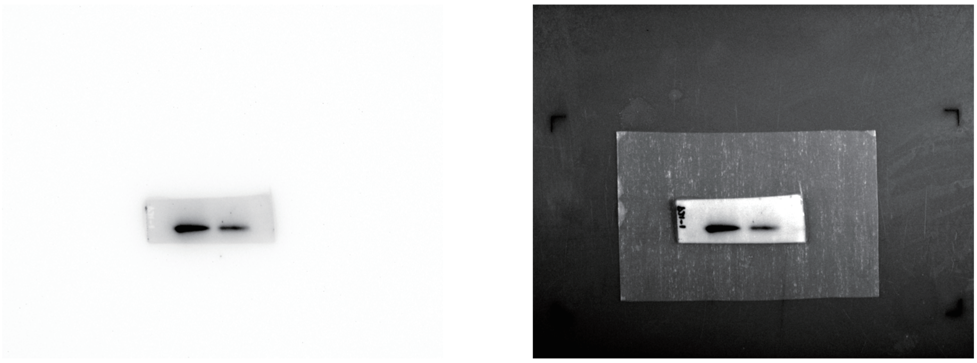

p53

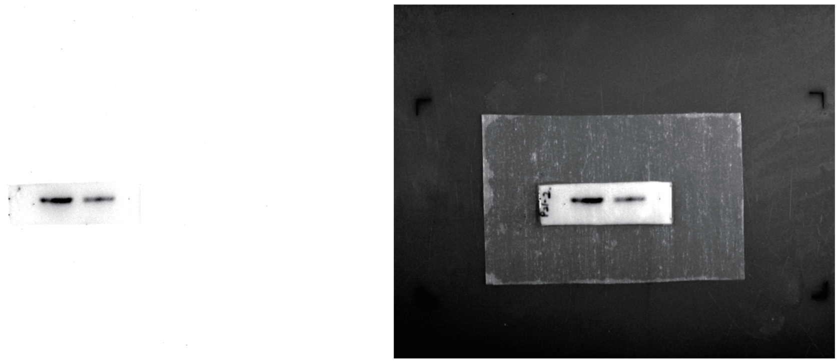

GAPDH

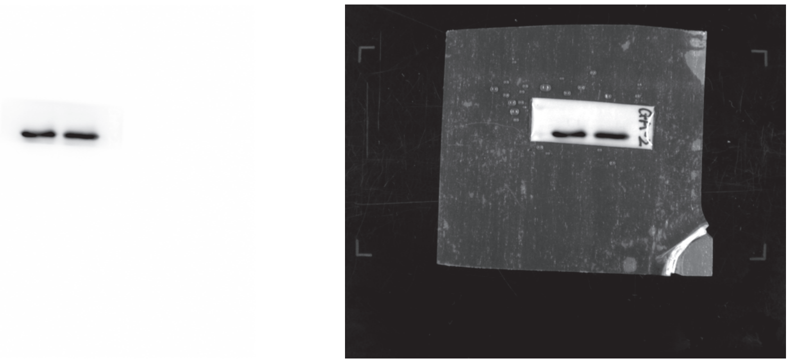

F2-J

PARP

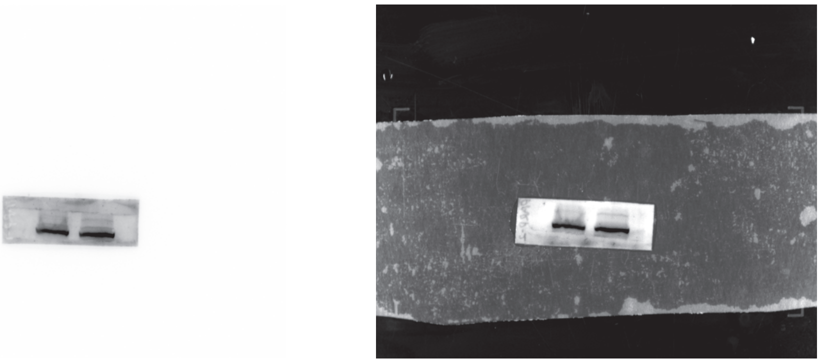

F2-L

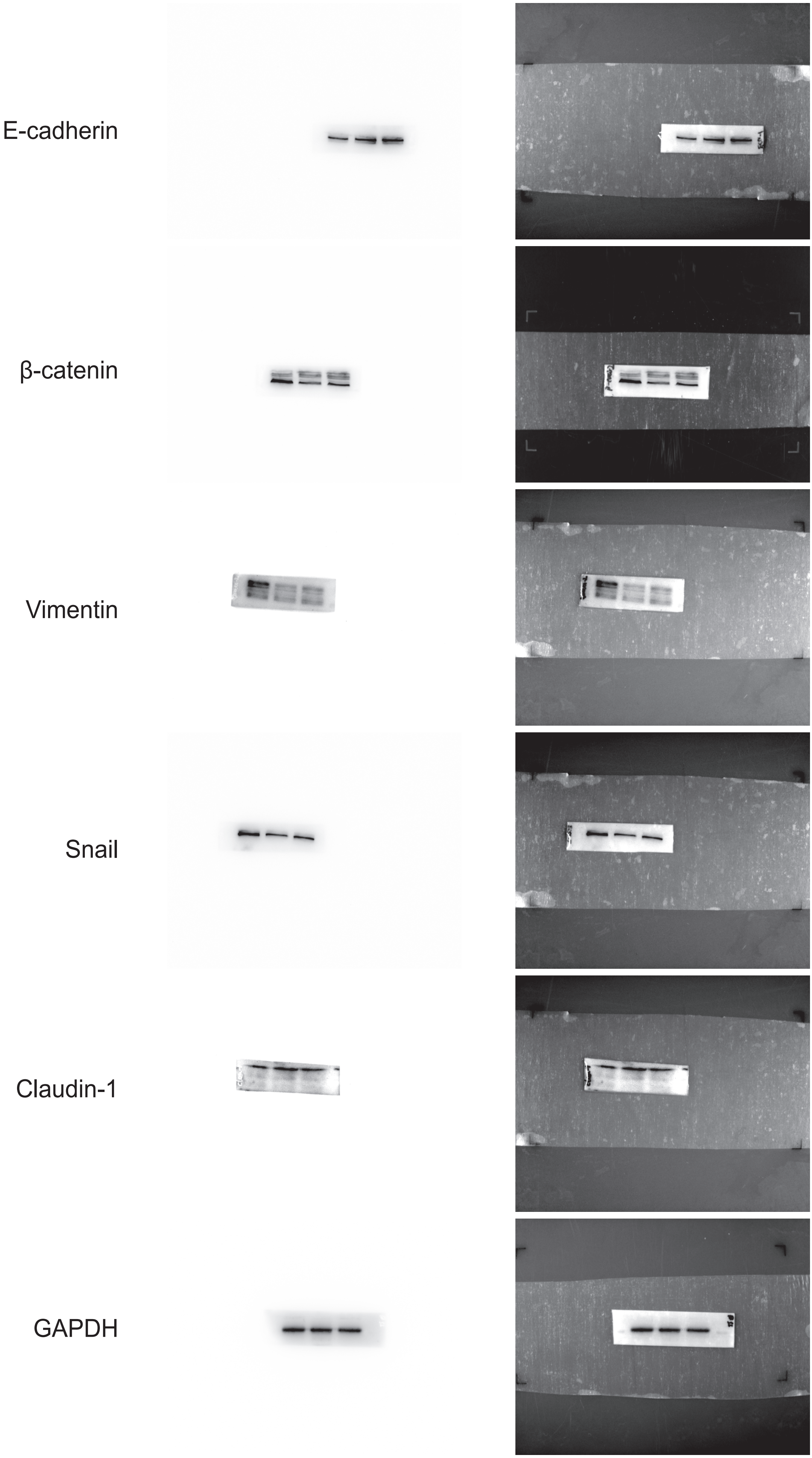

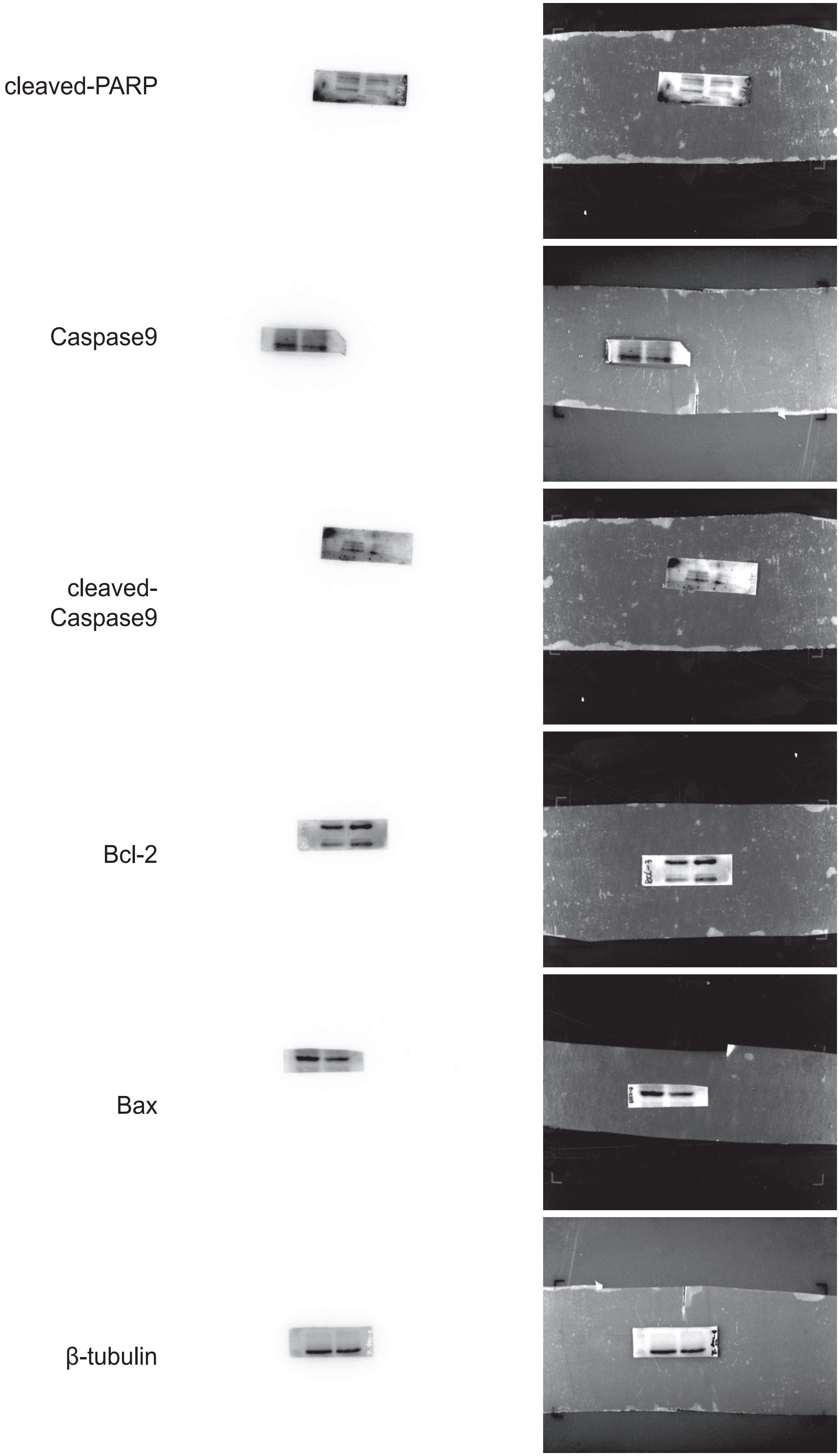
cleaved-PARP
Caspase9
cleaved-
Caspase9
Bcl-2
Bax
β-tubulin

F2-L

E-cadherin

β-catenin

Vimentin

snail

Claudin-1

GAPDH

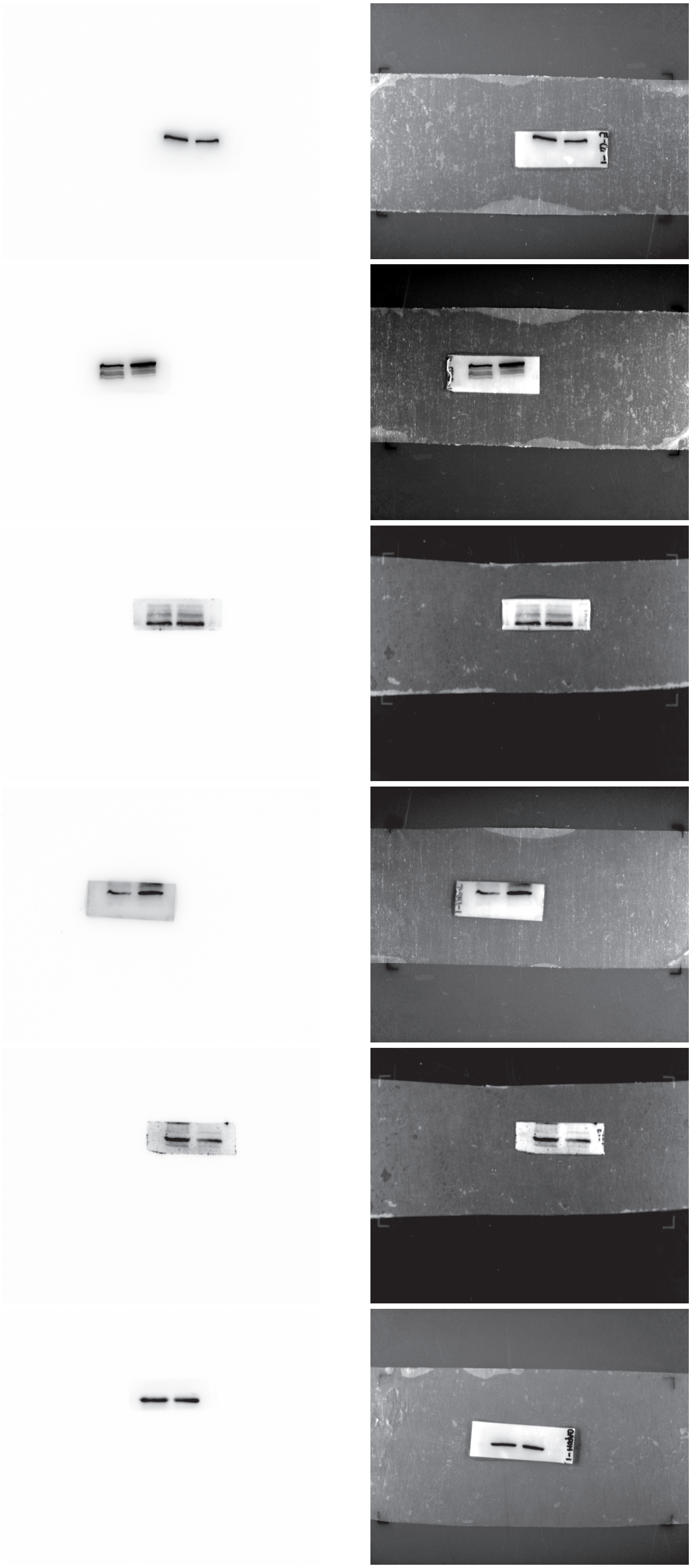

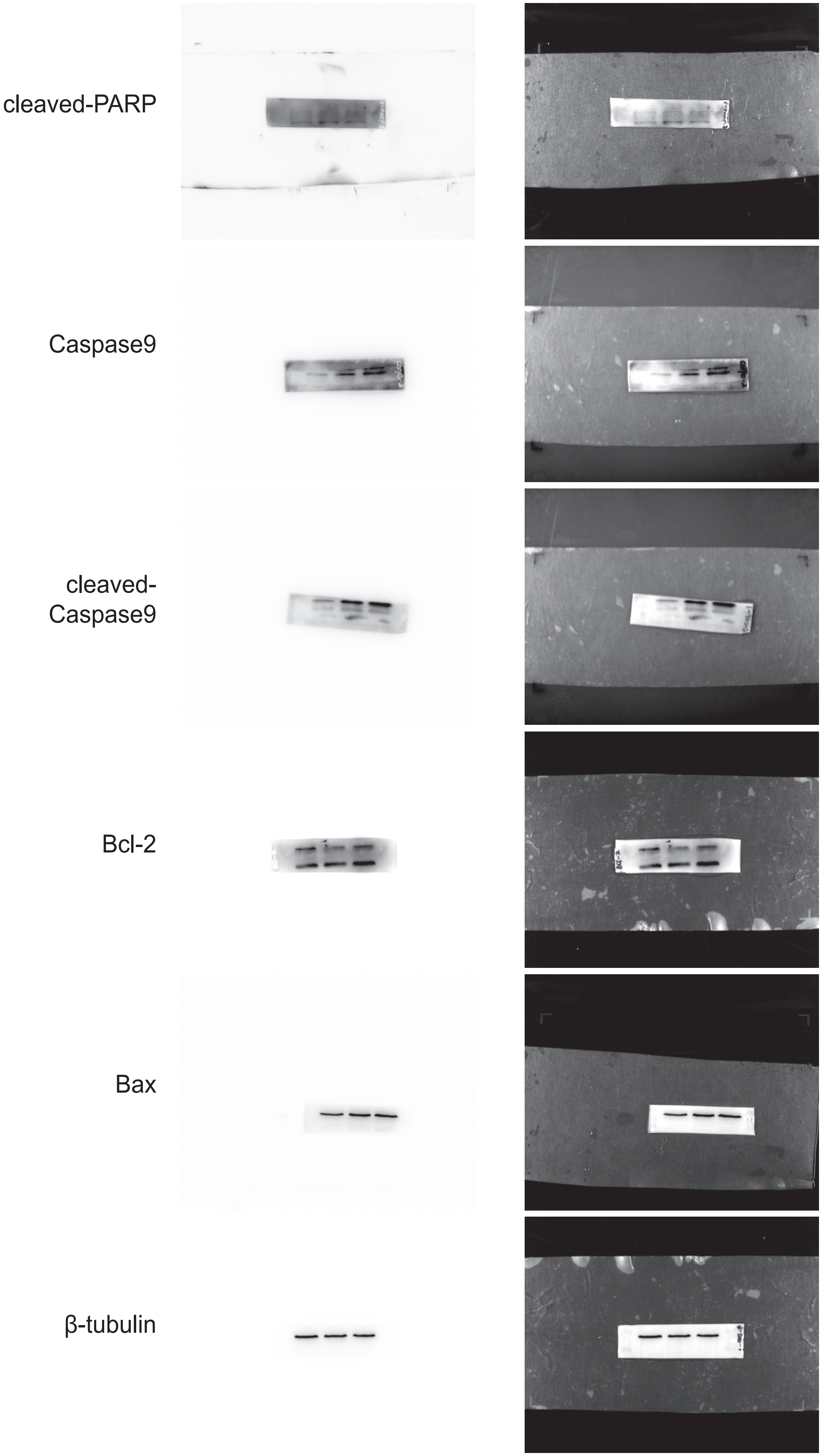
cleaved-PARP
Caspase9
cleaved-
Caspase9
Bcl-2
Bax
β-tubulin

F5-H

C4-2-sicirc-ETS1

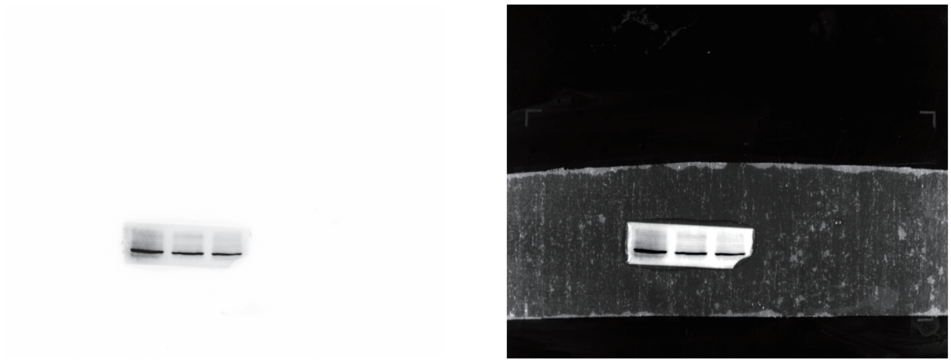

C4-2-sicirc-GAPDH

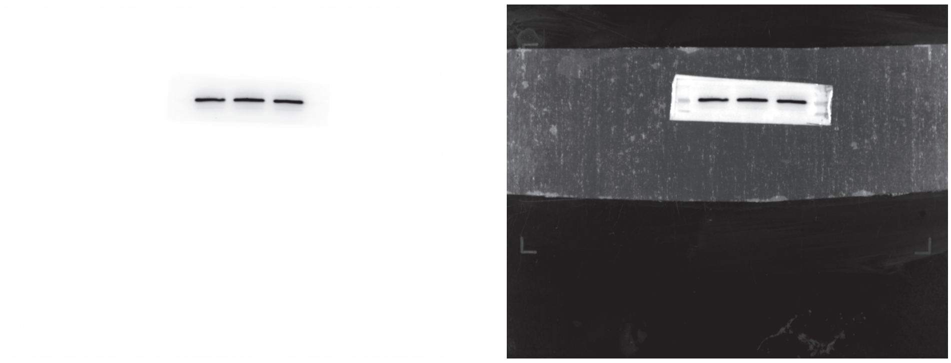

LNCaP-sicirc-ETS1

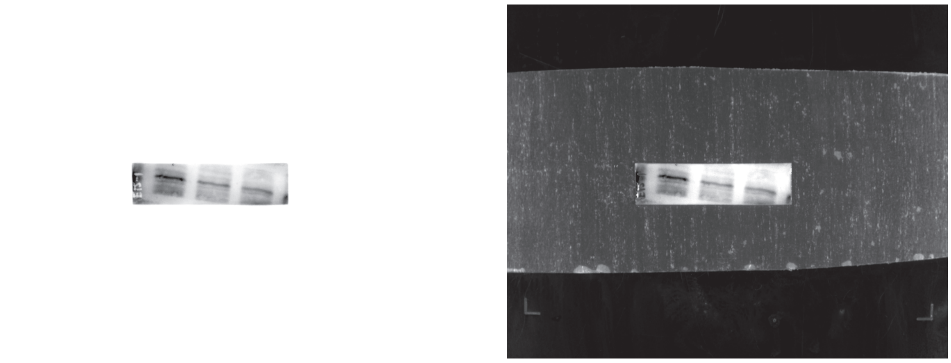

LNCaP-sicirc-GAPDH

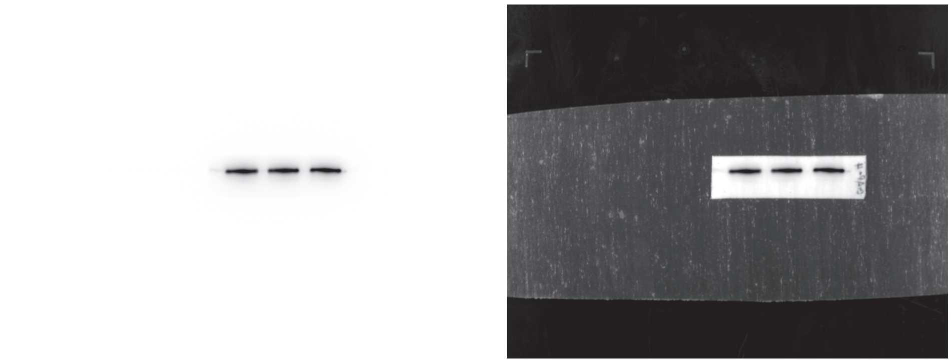

C4-2-lvcirc-ETS1

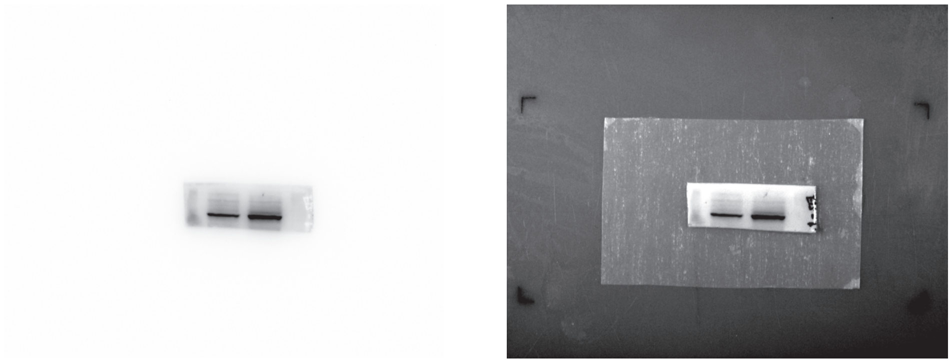

C4-2-lvcirc-GAPDH

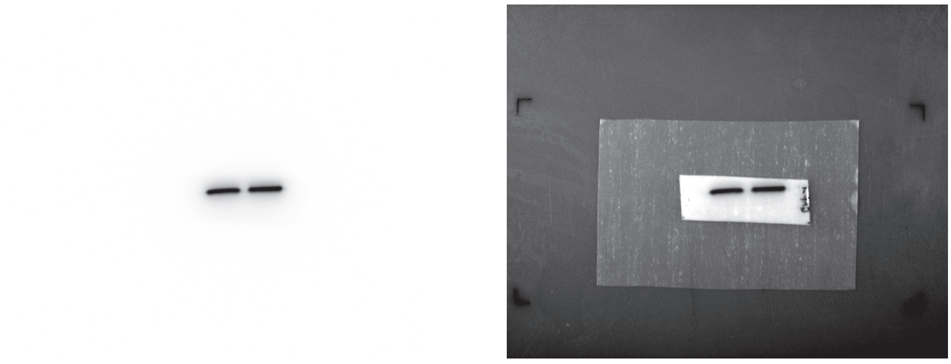

LNCaP-lvcirc-ETS1

LNCaP-lvcirc-GAPDH

F5-K

ETS1

Bcl2

Bax

GAPDH

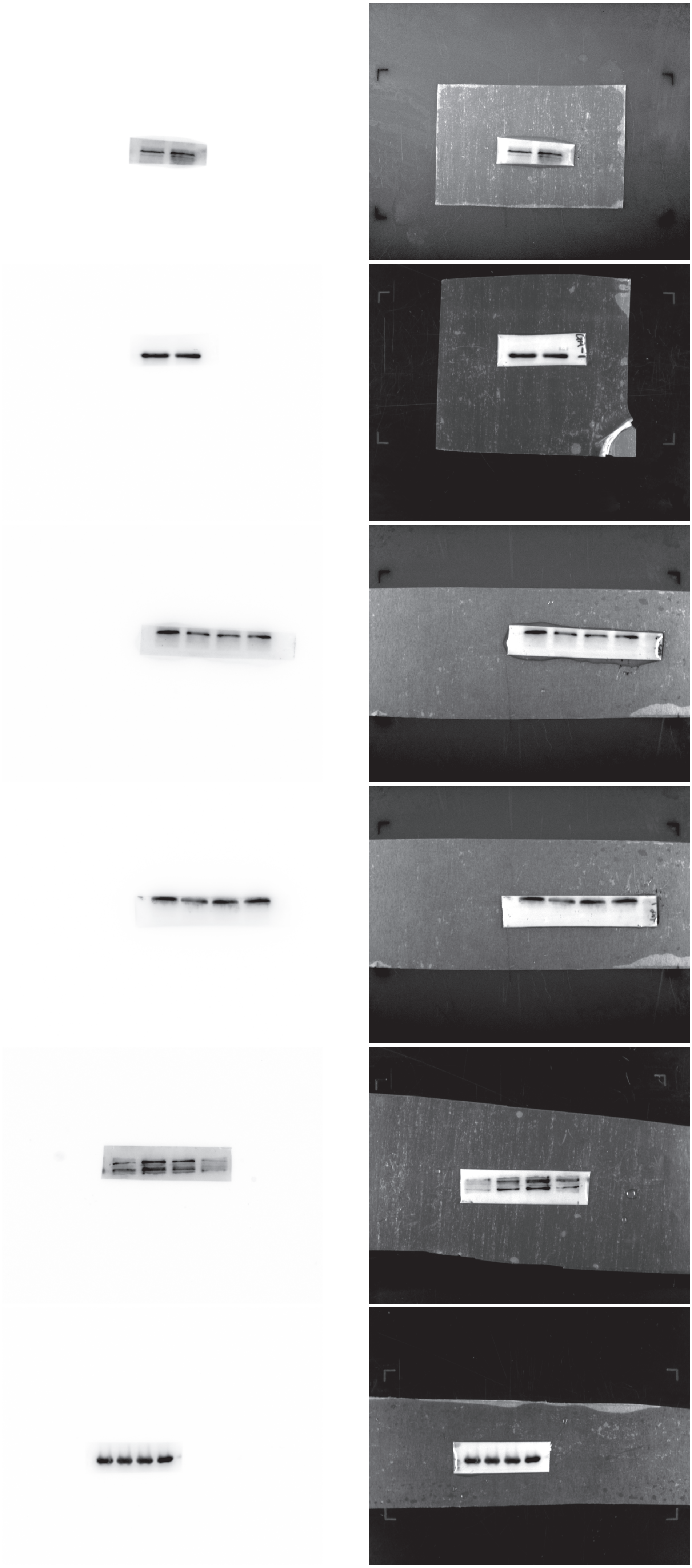

ETS1

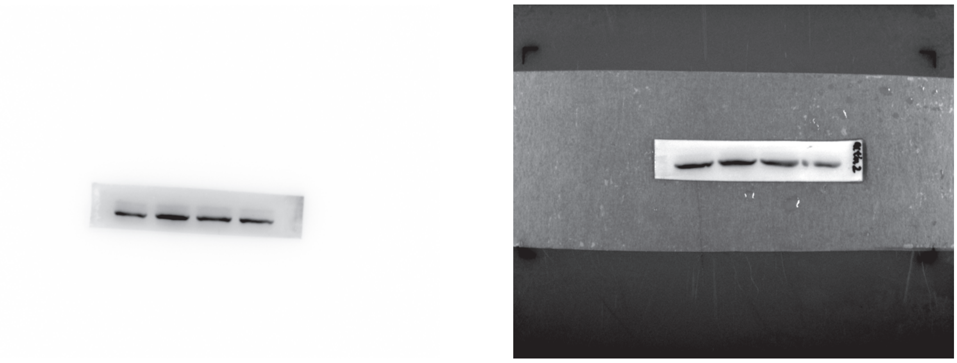

Bcl2

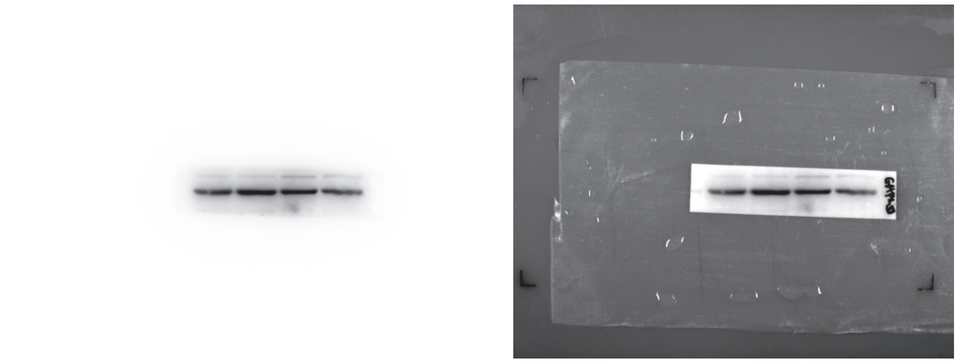

Bax

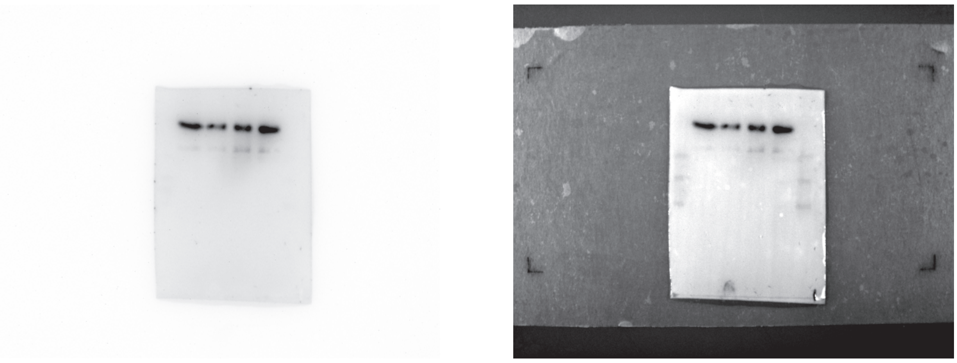

α-Tubulin

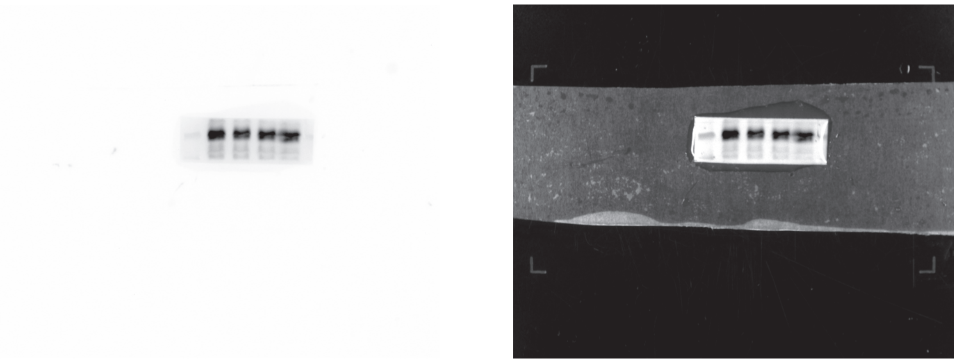

F5-L

ETS1

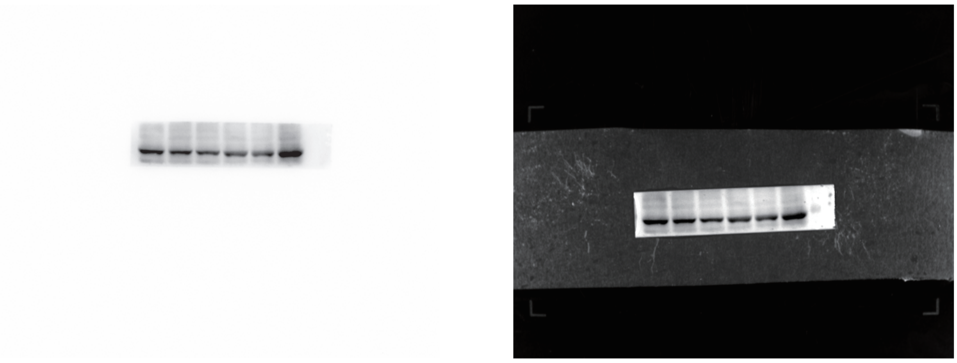

Bcl2

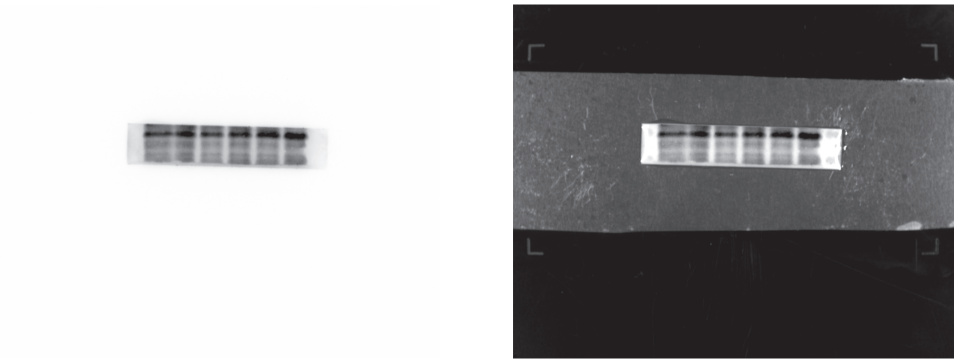

Bax

GAPDH

ETS1

Bcl2

Bax

GAPDH

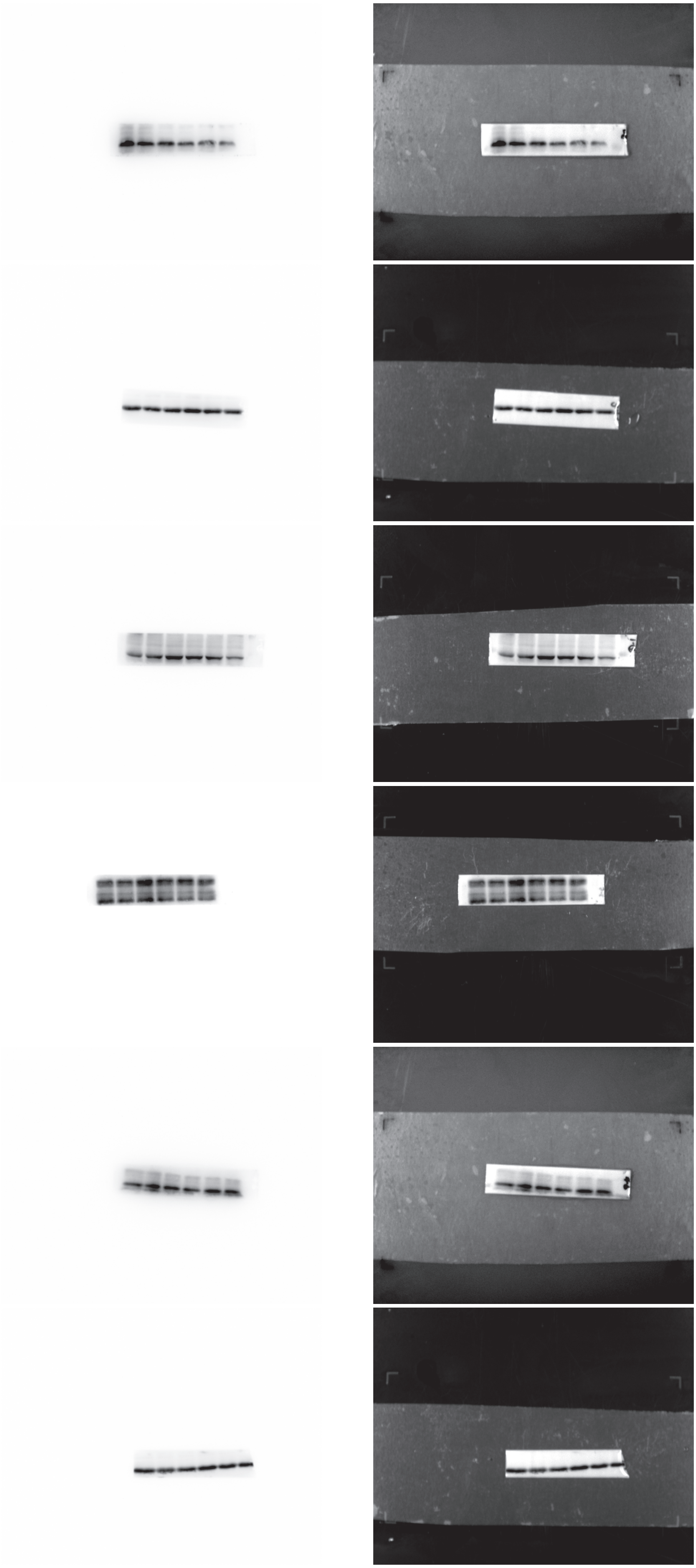

F6-D

ETS1

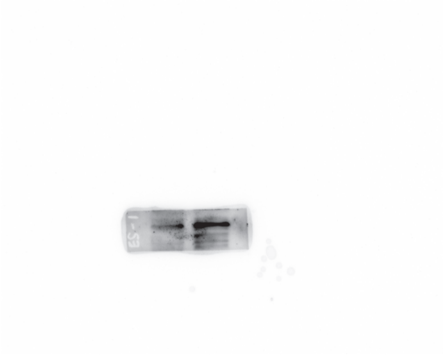

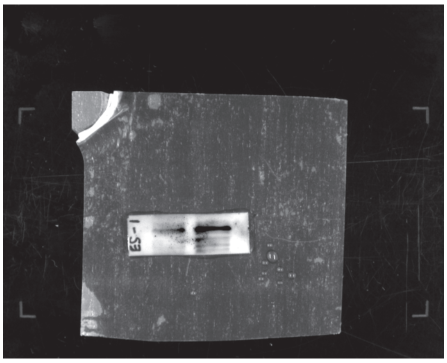

GAPDH

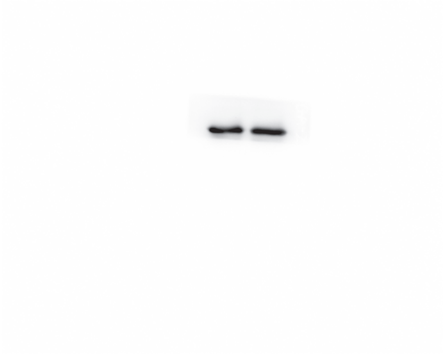

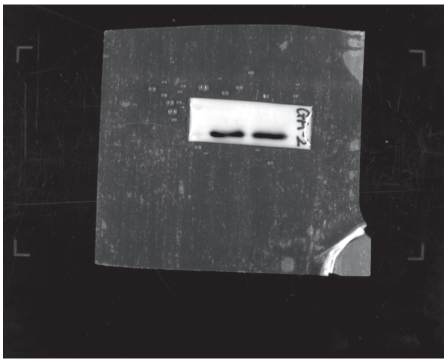

SF1-D

HNRNPL

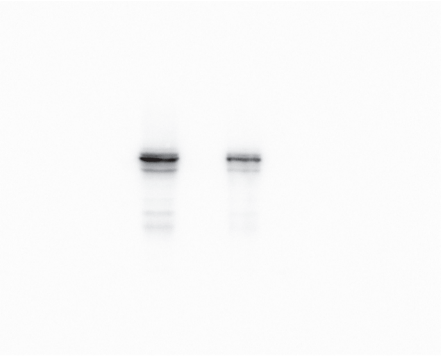

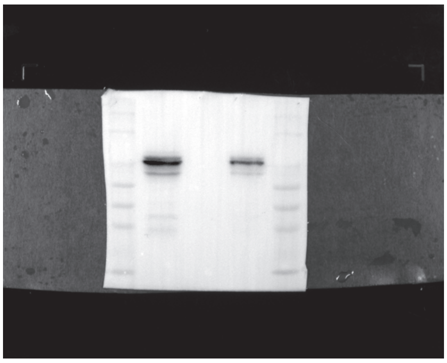

GAPDH

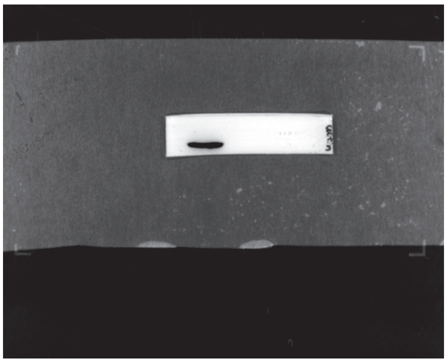

SF2-E

CyclinD1

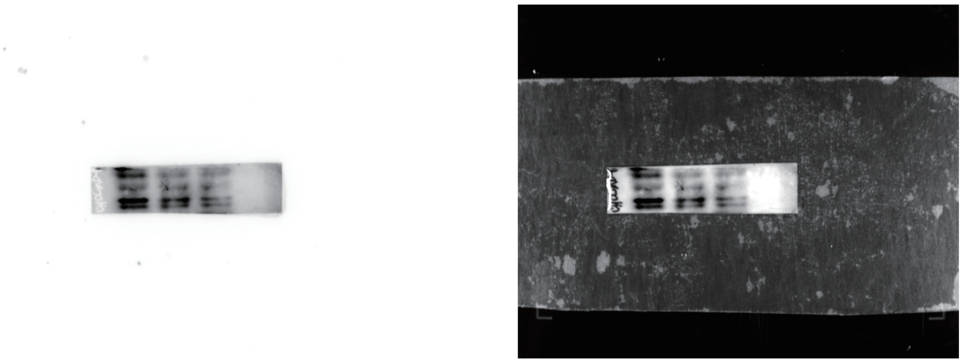

CDK4

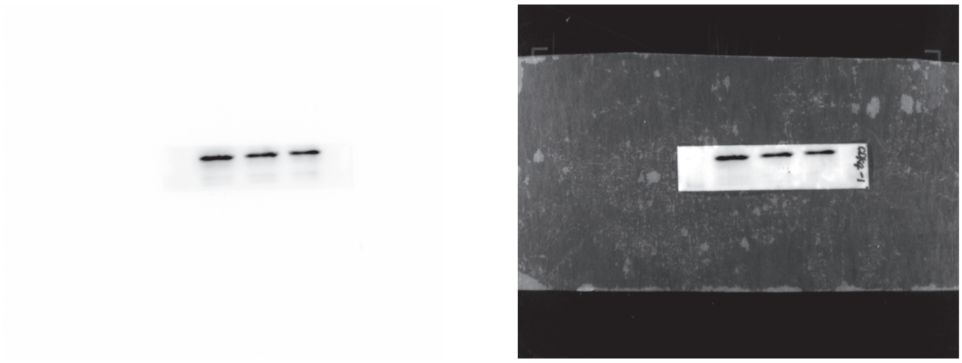

p21

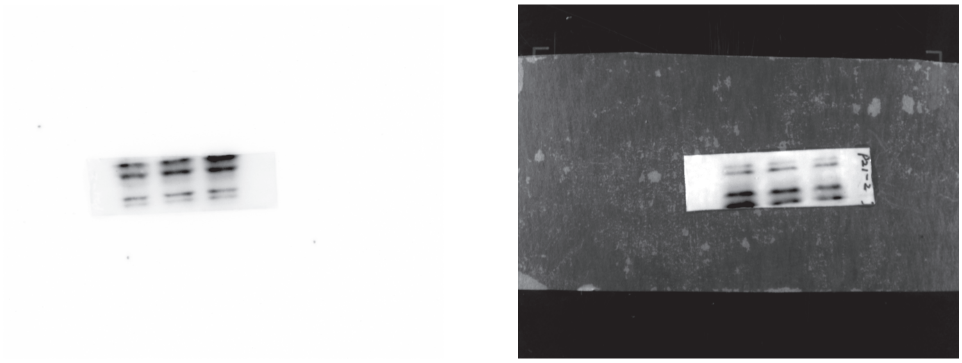

p53

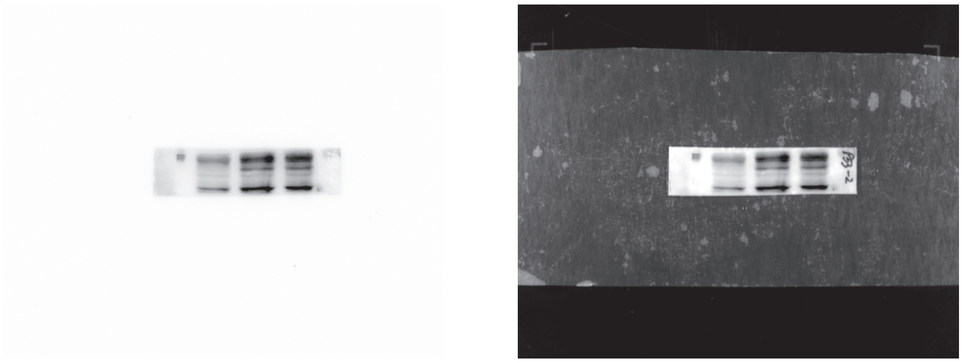

GAPDH

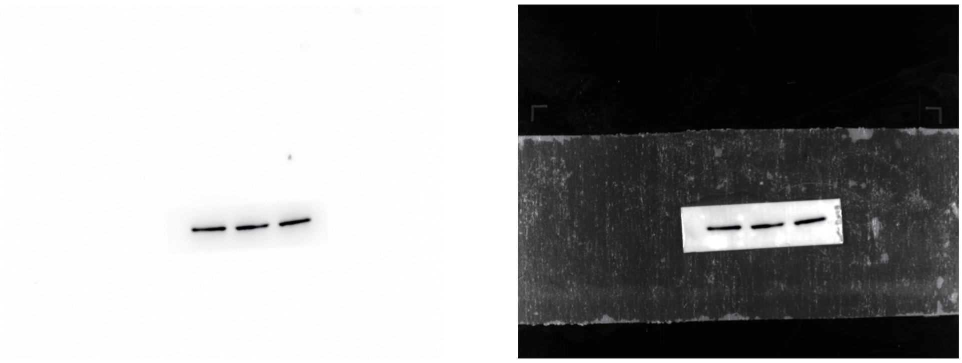

SF2-G

PARP

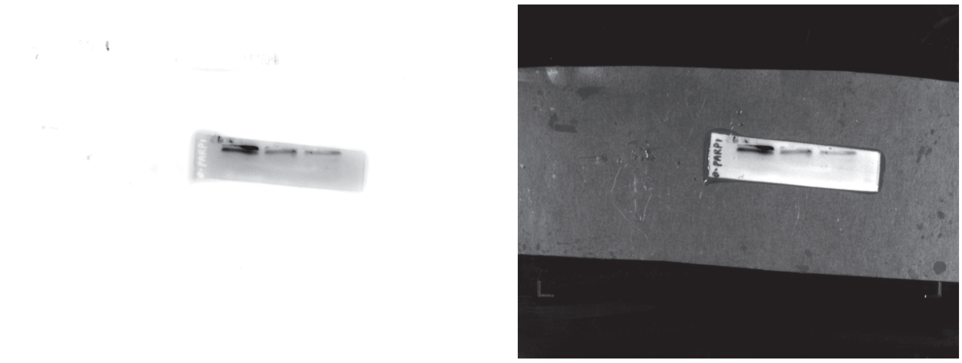

SF2-I

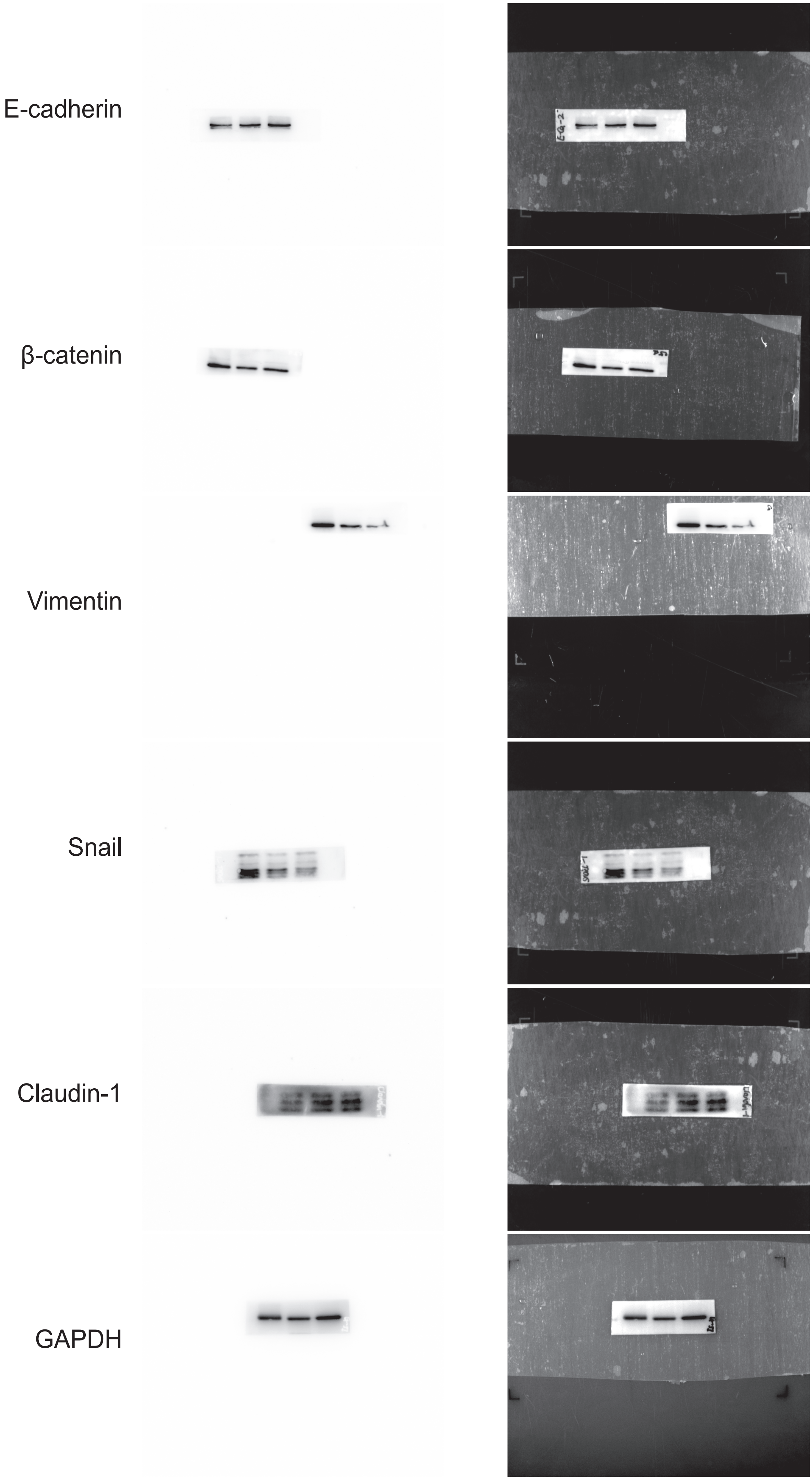

SF2-E

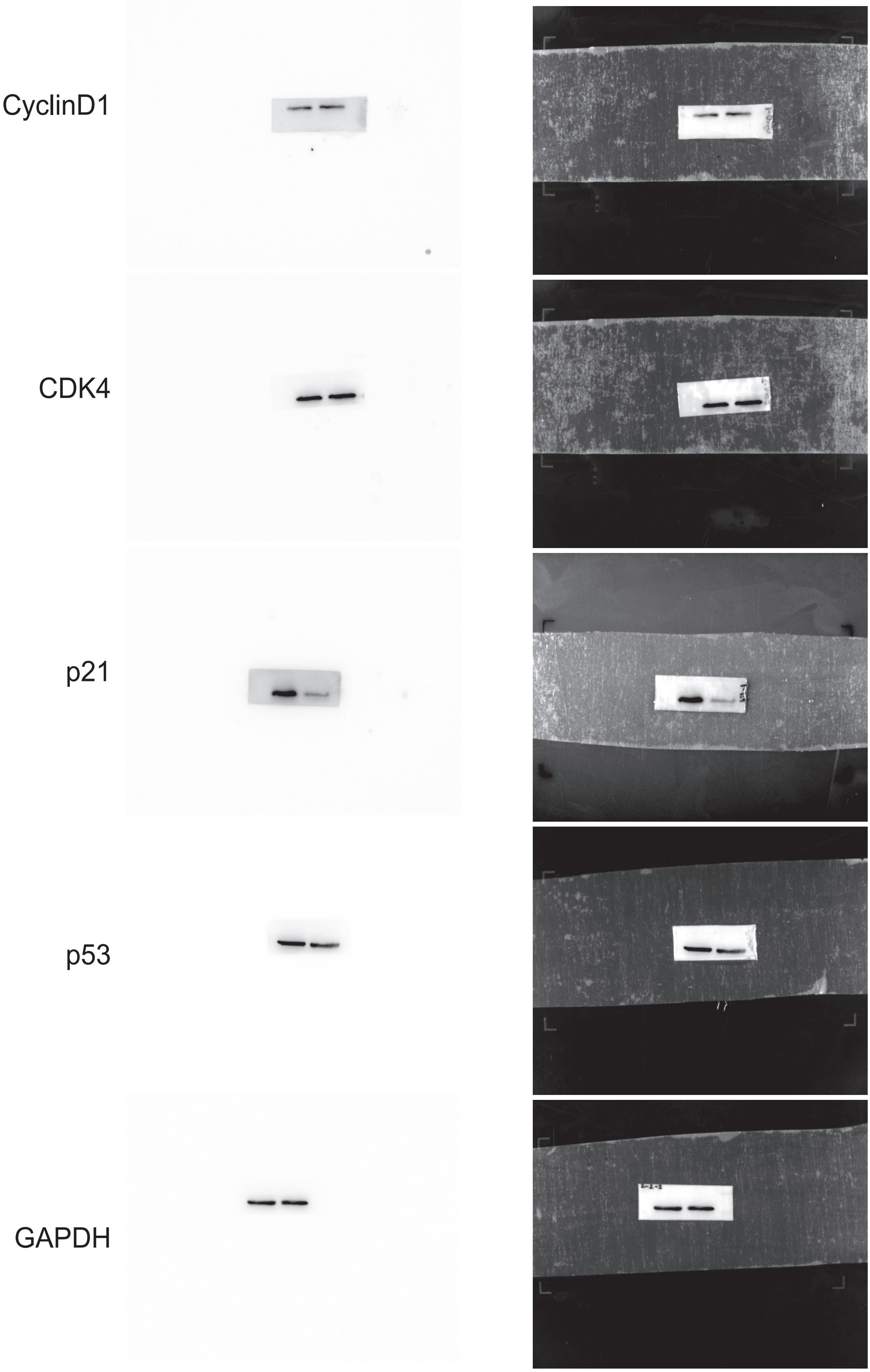

SF2-G

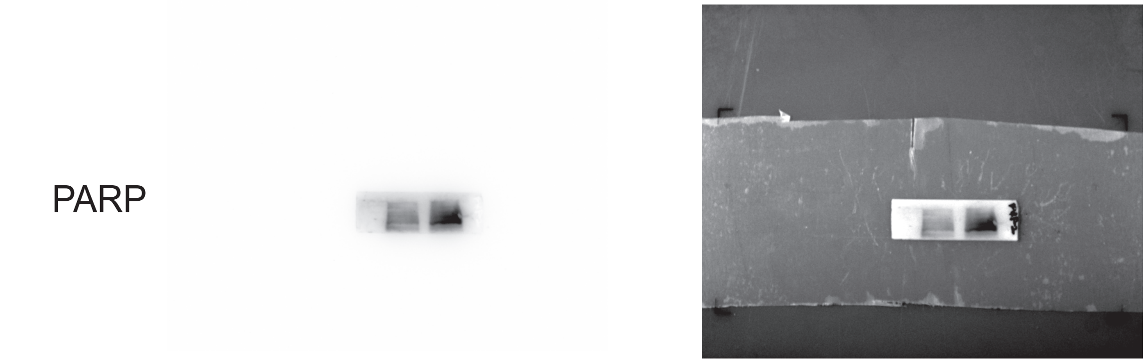

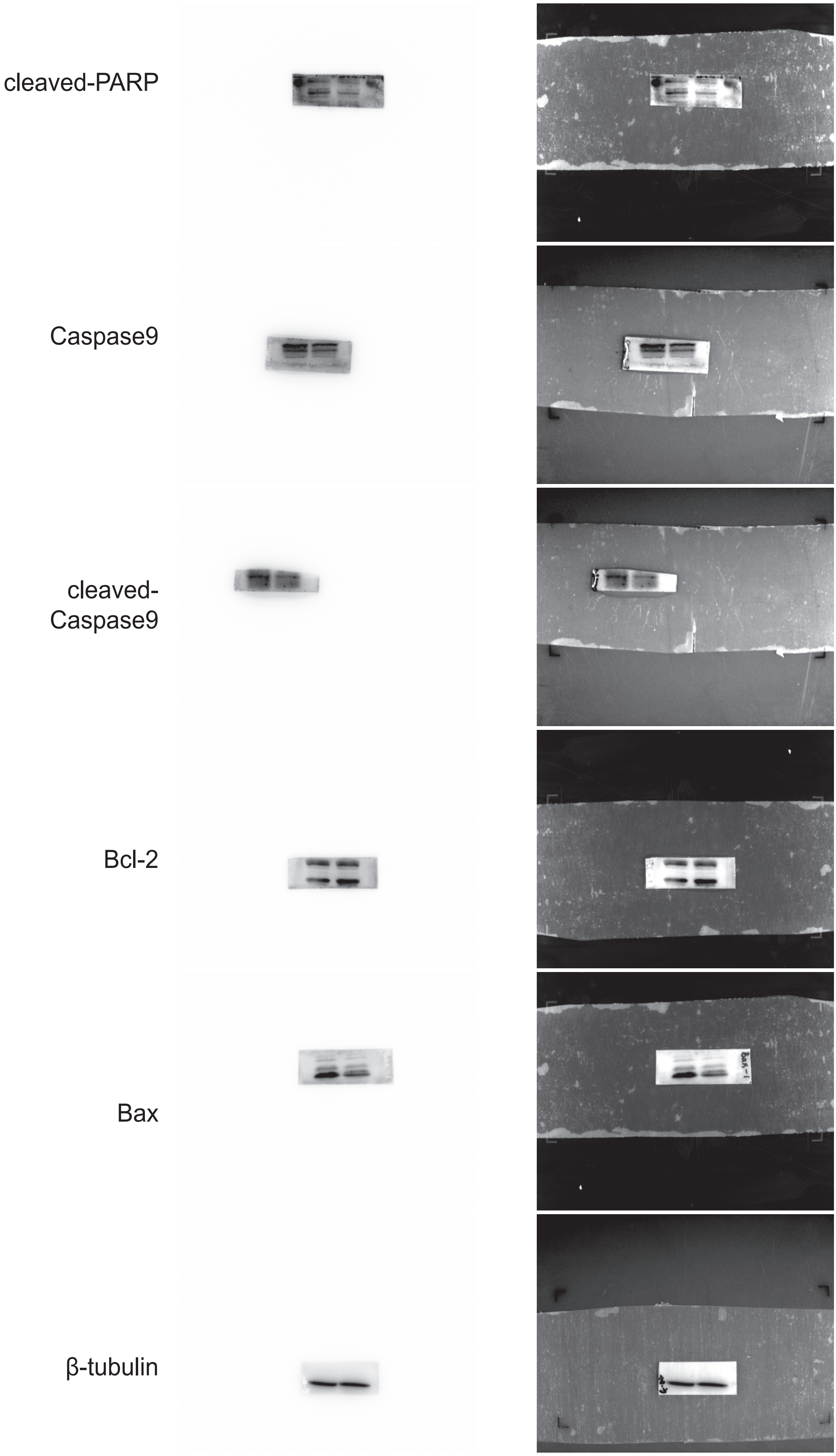
cleaved-PARP
Caspase9
cleaved-Caspase9
Bcl-2
Bax
β-tubulin

SF2-I

E-cadherin

β-catenin

Vimentin

snail

Claudin-1

GAPDH

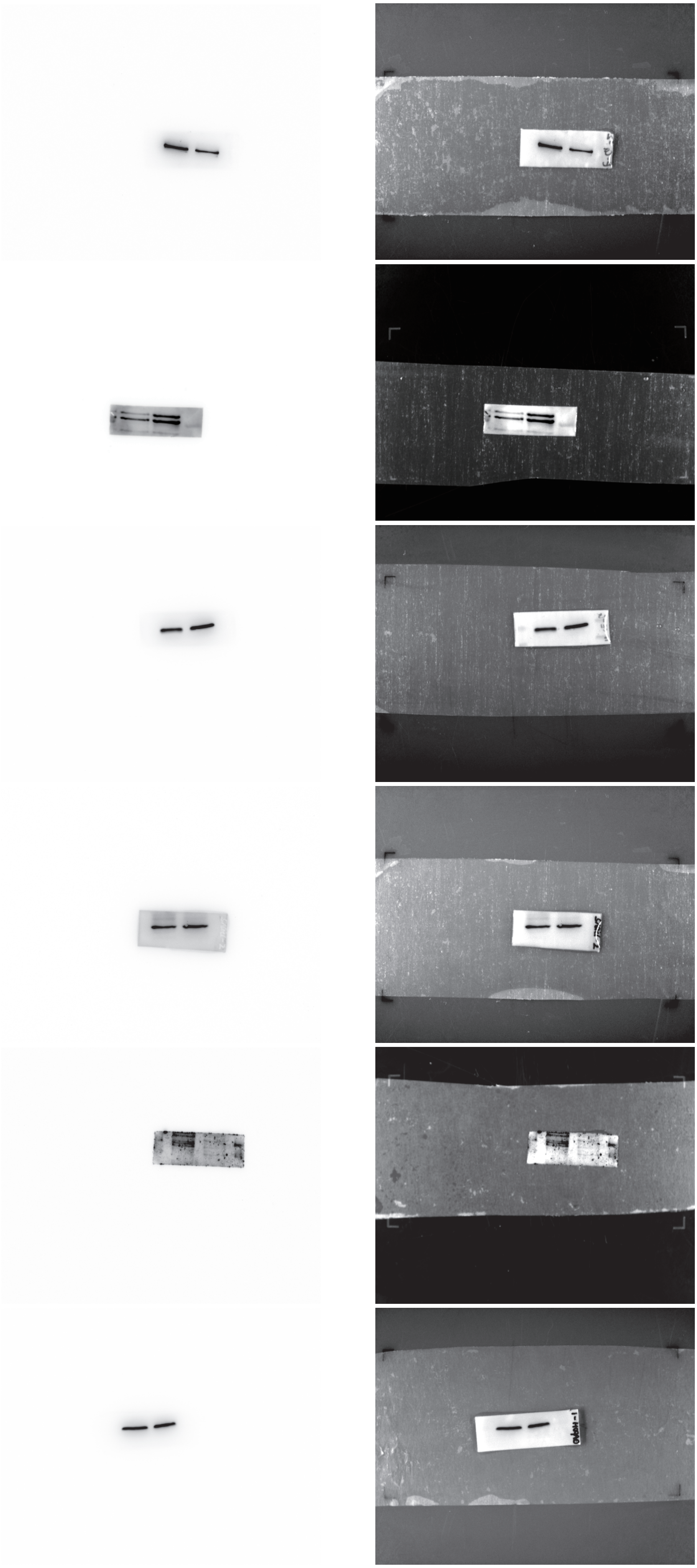

SF5-D

C4-2-si-ETS1

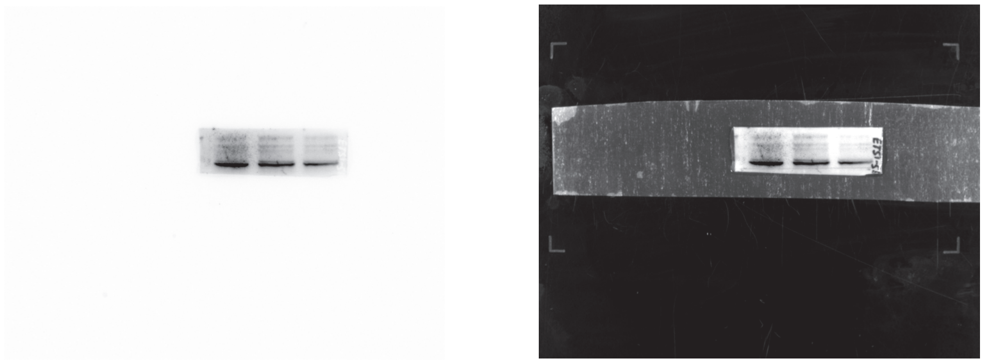

C4-2-si-GAPDH

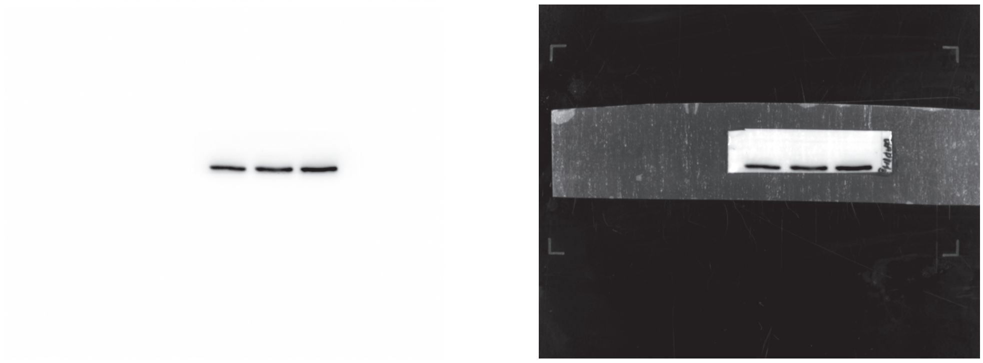

LNCaP-si-ETS1

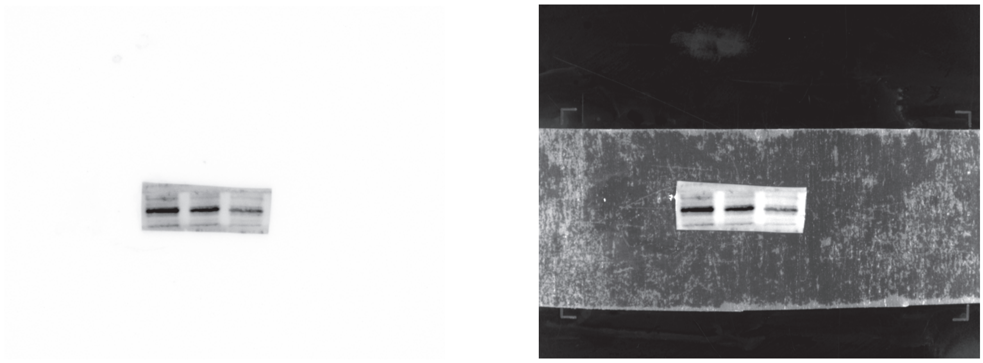

LNCaP-si-GAPDH

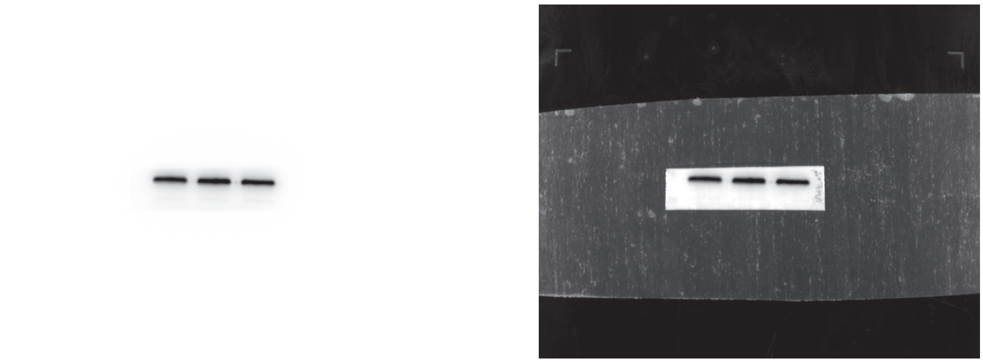

C4-2-lv-ETS1

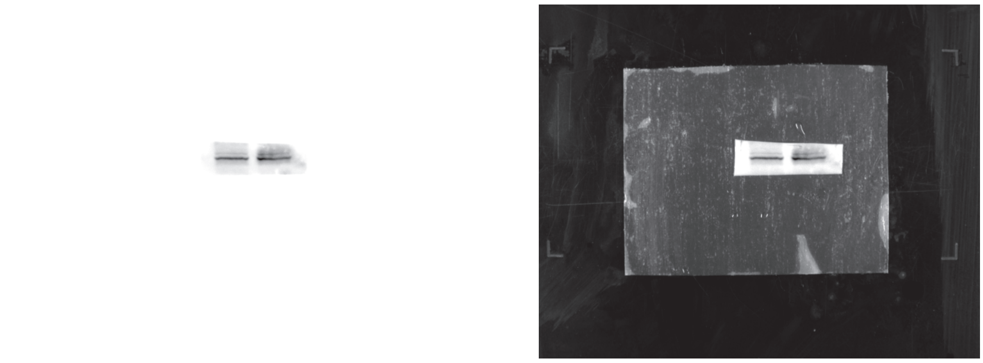

C4-2-lv-GAPDH

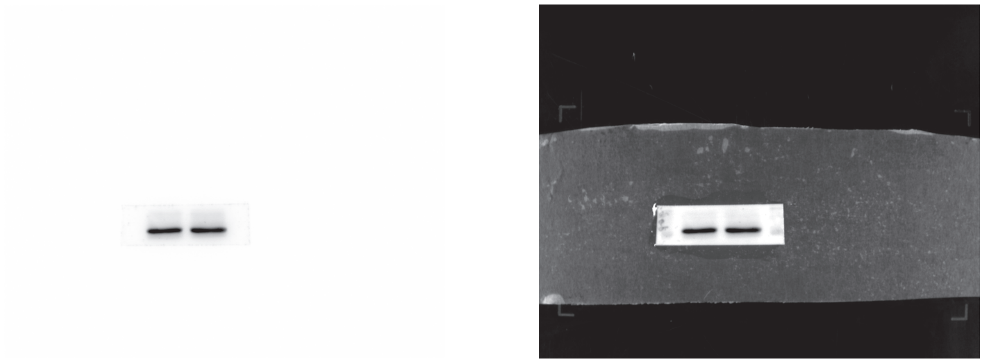

LNCaP-lv-ETS1

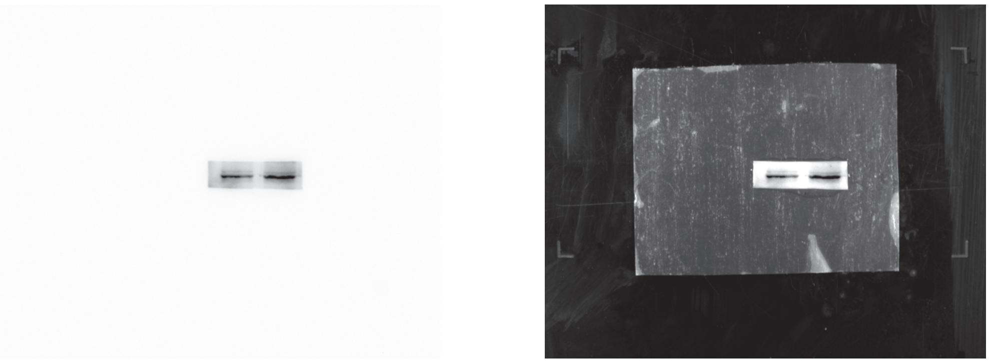

LNCaP-lv-GAPDH

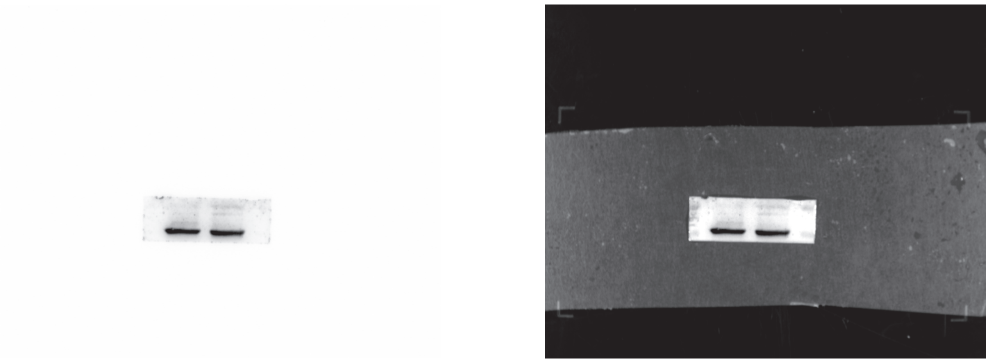

SF5-E

LNCaP-ETS1

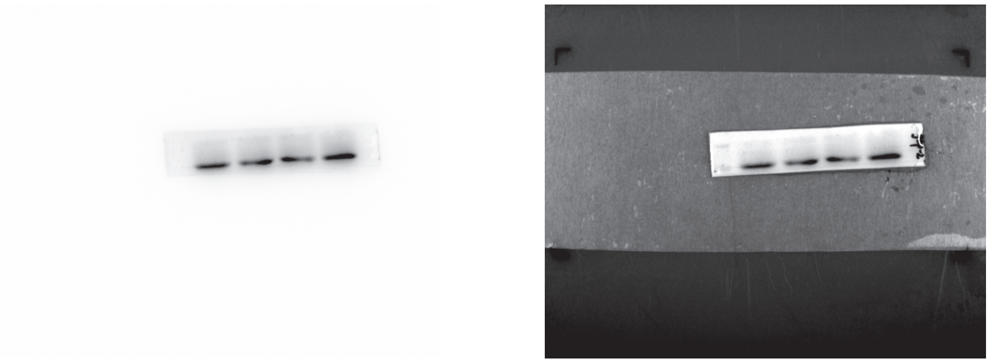

LNCaP-Bcl2

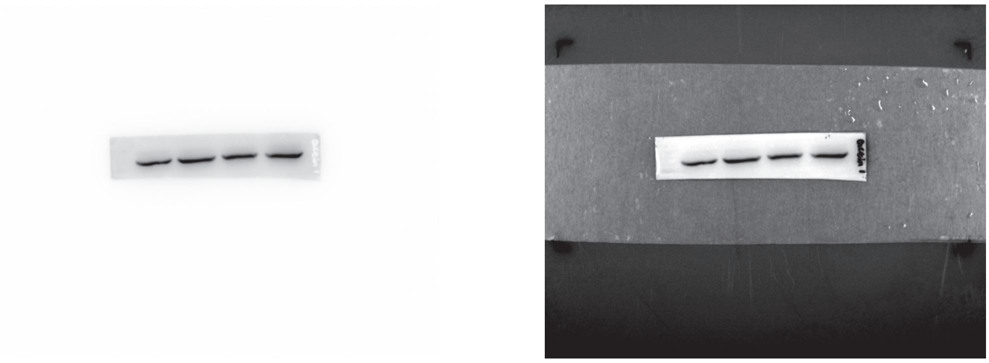

LNCaP-Bax

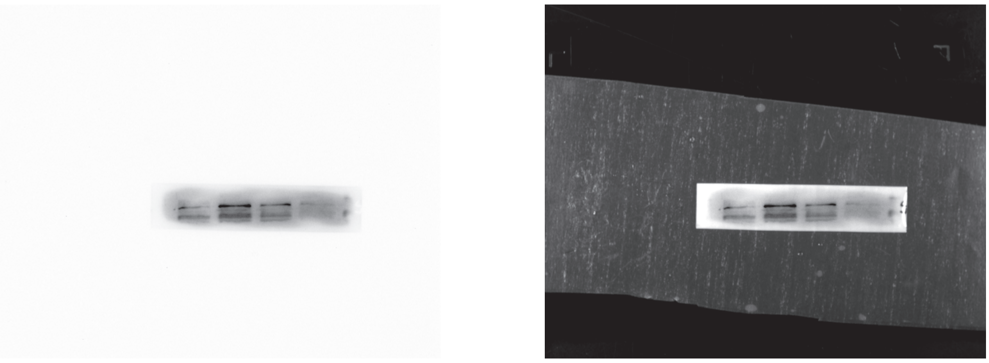

LNCaP-GAPDH

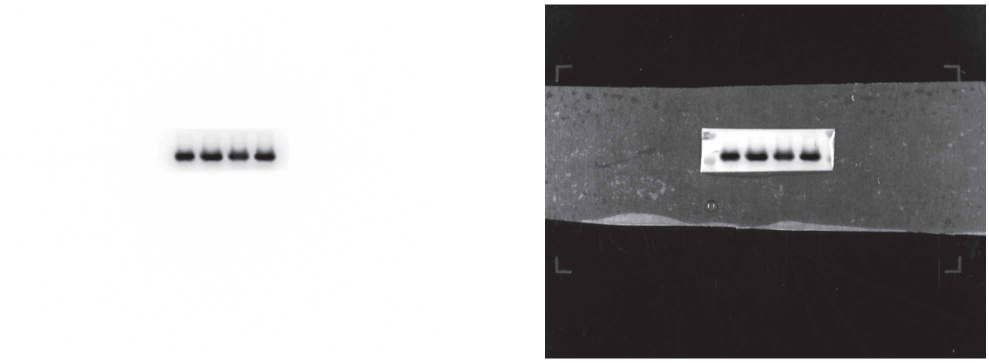

22RV1-ETS1

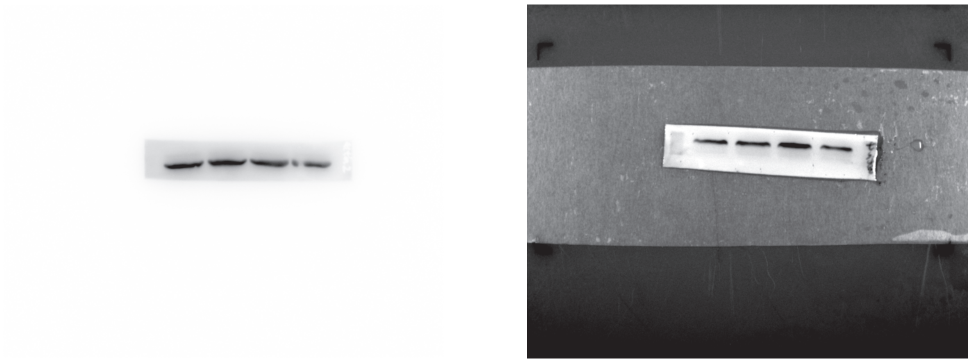

22RV1-Bcl2

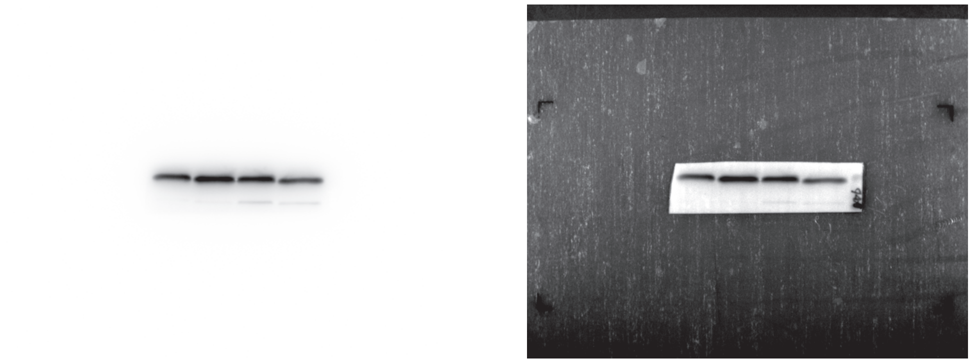

22RV1-Bax

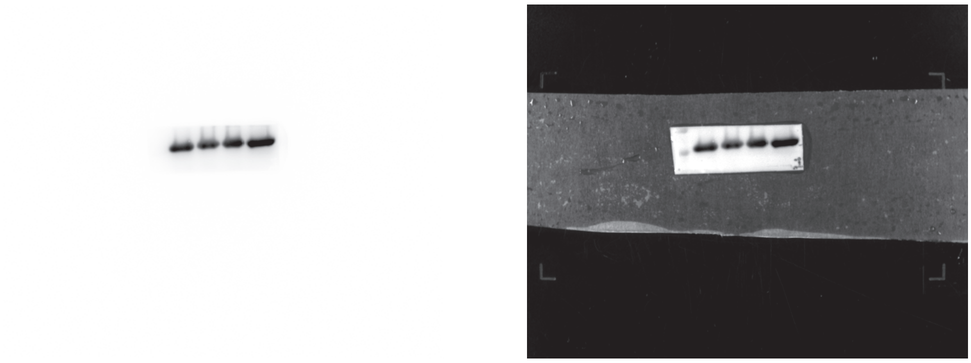

22RV1-α-Tubulin

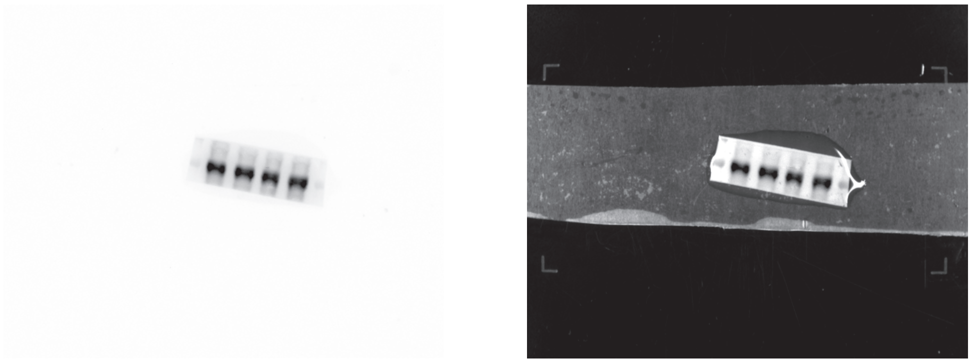

SF5-F

LNCaP-ETS1

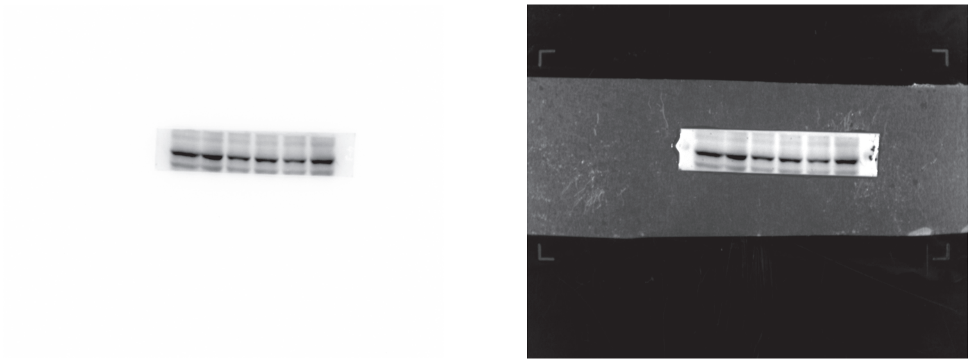

LNCaP-Bcl2

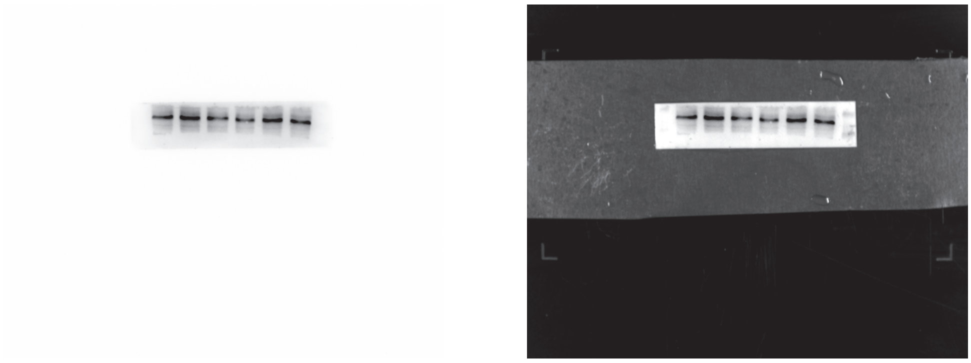

LNCaP-Bax

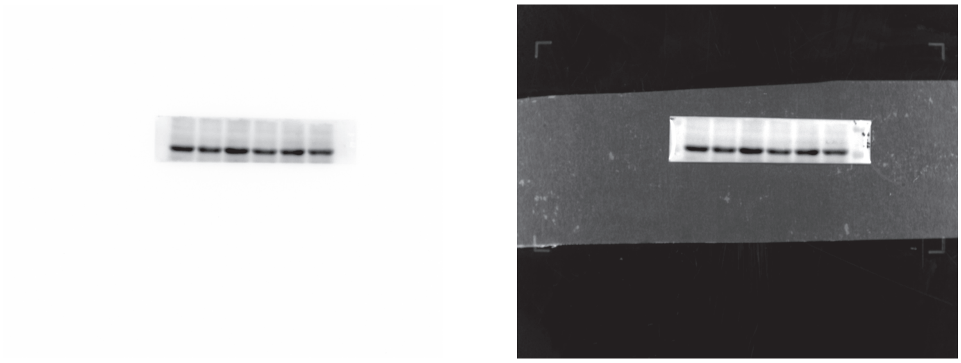

LNCaP-GAPDH

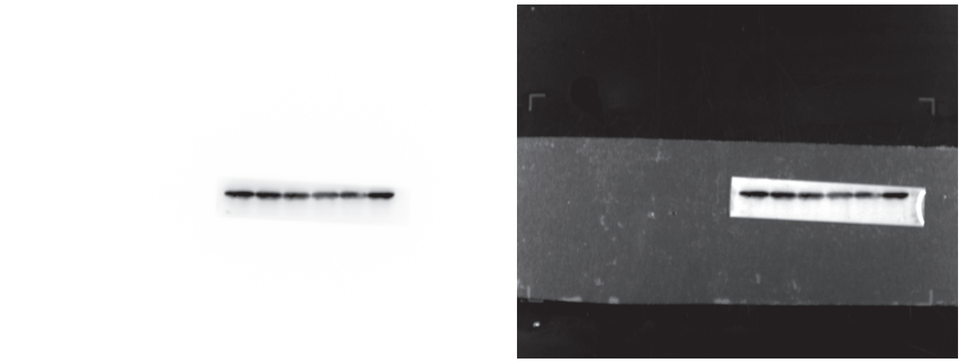

22RV1-ETS1

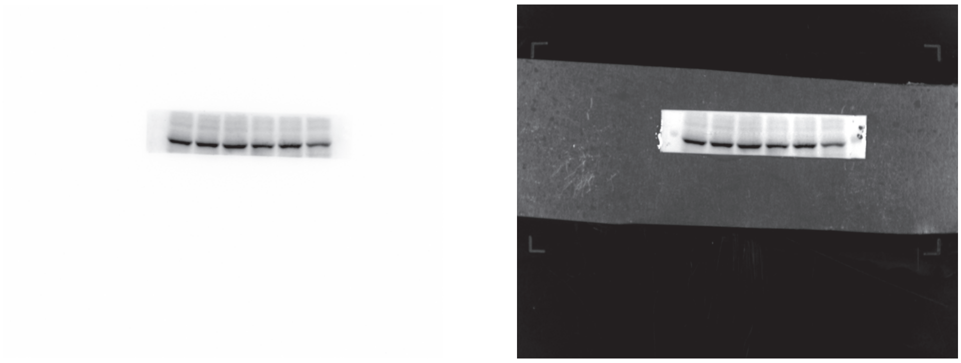

22RV1-Bcl2

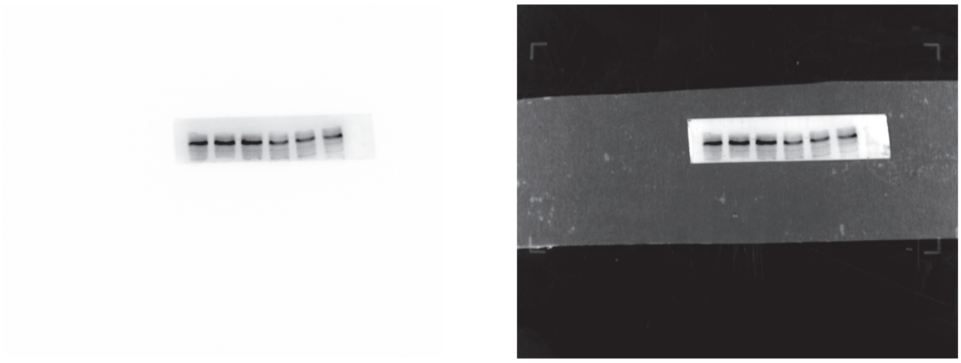

22RV1-Bax

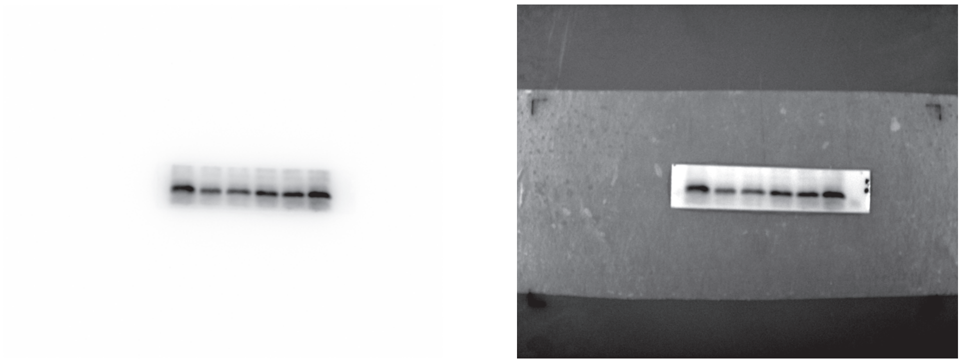

22RV1-GAPDH

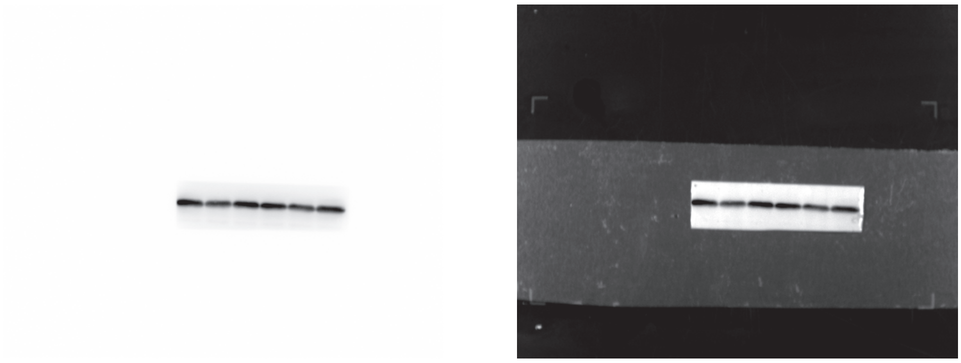

Supplement: Supplementary file 5 — Original Western Blot image [file 41420_2025_2904_MOESM5_ESM.pdf]
